# Drug Disposition in the Lower Gastrointestinal Tract: Targeting and Monitoring

**DOI:** 10.3390/pharmaceutics13020161

**Published:** 2021-01-26

**Authors:** Glenn Lemmens, Arno Van Camp, Stephanie Kourula, Tim Vanuytsel, Patrick Augustijns

**Affiliations:** 1 Drug Delivery and Disposition, KU Leuven, Gasthuisberg O&N II, Herestraat 49—Box 921, 3000 Leuven, Belgium; glenn.lemmens@kuleuven.be (G.L.); arno.vancamp@kuleuven.be (A.V.C.); 2 Drug Metabolism and Pharmacokinetics, Janssen R&D, Turnhoutseweg 30, 2340 Beerse, Belgium; skourula@its.jnj.com; 3 Translational Research Center for Gastrointestinal Disorders, TARGID, KU Leuven, Herestraat 49, 3000 Leuven, Belgium; tim.vanuytsel@kuleuven.be

**Keywords:** colonic physiology, colon drug delivery, colonic drug disposition, intestinal in vitro models, drug absorption, drug metabolising enzymes (DME), microbiome, microphysiological systems (MPS)

## Abstract

The increasing prevalence of colonic diseases calls for a better understanding of the various colonic drug absorption barriers of colon-targeted formulations, and for reliable in vitro tools that accurately predict local drug disposition. In vivo relevant incubation conditions have been shown to better capture the composition of the limited colonic fluid and have resulted in relevant degradation and dissolution kinetics of drugs and formulations. Furthermore, drug hurdles such as efflux transporters and metabolising enzymes, and the presence of mucus and microbiome are slowly integrated into drug stability- and permeation assays. Traditionally, the well characterized Caco-2 cell line and the Ussing chamber technique are used to assess the absorption characteristics of small drug molecules. Recently, various stem cell-derived intestinal systems have emerged, closely mimicking epithelial physiology. Models that can assess microbiome-mediated drug metabolism or enable coculturing of gut microbiome with epithelial cells are also increasingly explored. Here we provide a comprehensive overview of the colonic physiology in relation to drug absorption, and review colon-targeting formulation strategies and in vitro tools to characterize colonic drug disposition.

## 1. Introduction

Oral drug delivery is the most convenient application to target colonic diseases, due to its high patient acceptance and compliance compared to other application forms. Colon-targeted oral drug delivery should withstand the harsh gastrointestinal (GI) environment in order to release the drug in the colon, resulting in local tissue accumulation, low systemic absorption, and minimized adverse effects. Also, locally and systemically acting colon-targeted proteins and peptides (biologicals) are formulated to circumvent degradation by the low gastric pH and intestinal and digestive enzymes, so as to enable absorption by the colonic mucosa.

The pharmacokinetic disposition of a colon-targeting formulation is an intricate process. Firstly, upon reaching the colon, the drug release will depend on the selected release strategy: colon-targeted drug delivery systems are often engineered towards physiological colonic triggers such as the pH in the terminal ileum, the extensive transit time, degradation by the ubiquitous microflora, or are designed with physiology in mind, including mucoadhesives. Subsequently, the drug’s physicochemical properties (i.e., pKa, solubility, logD, chemical stability) and the colonic fluid variables (i.e., composition, residual volumes, motility) underlie the dissolution behavior. Prior to uptake, the drug will encounter various colonic barriers such as the gut microbiome, the thick bilayered mucus, and ultimately the colonic mucosa that expresses uptake-, and efflux transporters and drug metabolising enzymes (DME) in a region-dependent manner. To successfully select clinical drug candidates and prevent attrition in drug development, there is (1) a need to better understand the interplay between the drug delivery system, the physicochemical properties of the drug, and physiological variables; (2) a need for adequate in vitro dissolution assays and media (physiological buffers, biorelevant media, fecal slurries, etc.) that allow physiologically relevant dissolution kinetics; and (3) better screening assays that closely mimic intestinal function in vitro as to assess intestinal stability, permeation and accumulation. Traditional immortalized cell lines (e.g., Caco-2) are the most established systems for the prediction of intestinal absorption through permeability assessments, yet they lack expression of various metabolising enzymes (i.e., CYP3A4) and are subject to clone, passage and culturing variability [1]. The morphology and functional complexity of the intestine is maintained in excised tissue from preclinical species or humans, yet is sparingly used for studying intestinal absorption processes as viability is limited and tissues are difficult to obtain, costly, and ethically challenged. An emerging technique is the use of stem cell-derived systems (organoids, intestinal microtissues, Organ-on-a-Chip) that more closely resemble the molecular and cellular phenotype of human tissue than cell lines, since they can be cultured into a differentiated and polarized epithelium that contains multiple cell types and exhibit more relevant metabolic profiles [2,3]. Furthermore, stem cell-derived models can originate from different donor populations (e.g., healthy, diseased, malignant, geriatric, pediatric) and can be used to generate region-specific absorption and metabolism profiles. The permeation models discussed in this review represent colonic tissue, or small intestinal tissue for which a colonic variant is not yet available but can be foreseen. Moreover, colonic models incorporating commensal and pathogenic microbes are increasingly explored, as they allow the evaluation of microbiome-mediated drug metabolism [4,5] and permeability in one system; they could also be used to study the gut host–microbiome interaction by mimicking the interplay between the human gut microbiome, epithelial and immune cells if coculturing those three components could be achieved [6]. In this review, we provide a comprehensive overview of the colonic physiology in relation to drug absorption, formulation strategies, and the available in vitro tools to characterize GI drug disposition, with a focus on colonic absorption of small molecules (biologicals are out of the scope of this review).

## 2. Drug Barriers for Colonic Absorption

The colonic mucosa is a highly differentiated structure that comprises multiple cell types, which are localized at the surface or in the invaginations called crypts of Lieberkühn. The colonic crypts contain leucine-rich repeat-containing G-protein-coupled receptor 5 (LGFR5)-positive stem cells that give rise to transit-amplifying cells, which can rapidly proliferate, divide and differentiate into the various epithelial cell lineages [7]. The stem cells are often interspersed with CD24+ cells, and Paneth cells in the right hemicolon. Paneth cells are crucial for secreting antimicrobial peptides and providing the stem cell niche and might be represented by CD24+ cells [8,9]. Enteroendocrine L-cells, situated at the base of the crypt, produce hormones such as Glucagon-like peptide 1 and 2 (GLP-1/2) [10]. Differentiated colonocytes are mostly present at the surface of the crypt, and are important for resorption, secretion and humoral immunity. Furthermore, M-cells function as specialized surface epithelial cells that cover the lymphoid follicles of Peyer’s patches, and transport antigens and microorganisms to the underlying lamina propria, a connective tissue layer harboring the immune cells [11]. The amount of epithelial goblet cells ranges from 4% in the duodenum to 16% in the distal colon [12,13]; they produce a thick two-layered mucous intestinal lining, with the outermost layer colonized by a highly dense and diverse microbiome [14,15], and in contact with a complex luminal environment (presence of bile salts, lipid degradation products, etc.). The inner layer is more densely structured and largely devoid of bacteria [16]. 

### 2.1. The Colonic Epithelium

Intestinal models should represent the biochemical (metabolism, efflux, uptake) and physical (cell layer, mucus, unstirred water layer) barrier characteristics of the GI tract to enable an accurate prediction of intestinal absorption and metabolism. To this end, in vitro models need to accurately capture the different transport mechanisms, both passive and active. Passive diffusion is gradient driven and can occur paracellularly for low molecular weight hydrophilic molecules. It is determined by tight junctions, which are the most apical part of the junctional complexes that connect neighboring epithelial cells [17,18,19]. Paracellular transport is limited [20] as the available surface area is considerably lower (estimated at 1%) compared to transcellular transport. Not surprisingly, a substantial fraction of the commercially available drugs, 85–90%, permeate transcellularly [21], with lipophilic drugs generally partitioning into the cell membrane by passive diffusion. The absorption of drugs can also be facilitated by transporters or can undergo efflux out of the enterocytes. This process is often associated with drug–drug interactions (DDI) given its stereoisomer specific, saturable, and inhibitable nature [22]. Since the mucosa is functionally distinct throughout the GI tract, drug absorption can differ along the intestinal region. The absorption in the colon is compromised by various factors in comparison to the small intestine. First, the absence of villi in the colon drastically reduces the available surface area (from 60 m^2^ in the jejunum and ileum to 0.3 m^2^ in the colon) for passive absorption [23]. Secondly, the electrical resistance of the colonic epithelium, a measure for paracellular permeability, is higher than in the small intestine [17,18,19] resulting in decreased paracellular passive absorption. Furthermore, in humans, the left hemicolon is more permeable than the right hemicolon [24]. The regional expression of claudins, cell–cell adhesion molecules of the tight junctions, is likely at the base of these absorption differences [25]. Lastly, transporters and metabolizing enzymes are expressed in a manner specific to the colonic region, affecting drug absorption and metabolism. 

The absorption of a drug can be influenced by the activity of uptake and efflux transporters as well as metabolizing enzymes. Efflux transporters such as P-glycoprotein (P-gp; ATP binding cassette subfamily B member 1 (*ABCB1*)) have a higher affinity, especially for cationic lipophilic components to prevent a toxic build-up of harmful xenobiotics (e.g., alkaloids and flavonoids), whereas uptake transporters such as peptide transporter protein 1 (PEPT1; Solute carrier family 15 member 1 (*SLC15A1*)), monocarboxylate transporter protein 1 (MCT1; *SLC16A1*), and apical sodium-dependent bile acid transporter (ASBT; *SLC10A2*) take up hydrophilic compounds, e.g., dipeptides, monocarboxylic acids, and bile acids. These ionized and high molecular weight drugs generally cannot pass the epithelium transcellularly or paracellularly via passive diffusion. Although transporters have their respective roles in regulating drug absorption, the expression pattern is not homogenous along the GI tract [26,27].

In the colon, the following protein ratios were determined through a proteomics approach [26]: MCT1 (55%) > multidrug-resistance-associated protein (MRP3; *ABCC3*) (14%) > MRP4 (*ABCC4*) (9%) > MRP2 (*ABCC2*) (8%) > P-gp (5%) > organic anion transporting peptide 2B1 (OATP2B1; Solute carrier organic anion transporter family member 1B1 (*SLCO2B1*)) (4%) > breast cancer resistance protein (BCRP; ABCG2) (3%) > PEPT1 (2%) > organic cation transporter 1 (OCT1; *SLC22A1*) (1%). MCT1 and MRP4 are represented most highly in the colon, and MRP4 was homogenously present along the entire intestine. Some transporters displayed a gradientlike gene expression and protein abundance along the intestine. Efflux transporters P-gp and BCRP, and uptake transporter PEPT1 increased noticeably from the duodenum to distal jejunum and ileum, and substantially dropped in the colon. MRP2, MRP3 and OCT1 protein levels were highest in the jejunum. Various other transporters such as MRP1, Bile salt export pump (BSEP; *ABCB11*), multidrug and toxin extrusion 1 (MATE1; *SLC47A1*), Sodium/taurocholate cotransporting polypeptide (NTCP; *SLC10A1*), organic anion transporting polypeptide 2 (OAT2; *SLC22A7*) OATP1A2 (*SLCO1A2*), OATP1B1 (*SLCO1B1*), OATP1B3 (*SLCO1B3*), and OCT3 (*SLC22A3*) could not be detected by protein level. Trace amounts of *ABCC1* (MRP1), *SLC22A3* (OCT3), *SLC22A7* (OAT2), and *SLC47A1* (MATE1), were only observed in the form of messenger RNA (mRNA) [26].

Similar to transporter proteins, there is evidence that DMEs are expressed heterogeneously along the GI tract, with the highest expression levels in the proximal to mid small intestine, the main site of drug absorption and metabolism. The expression of Cytochrome P450 Family 3 Subfamily A Member 5 (CYP3A5), CYP2B6, CYP2J2 and particularly CYP3A4 and CYP2C9 is high in the duodenum and jejunum, but significantly decreases in the ileum/colon when quantified using liquid chromatography/tandem mass spectrometry [28]. This observation could be extended towards all the investigated DMEs, although CYP2C19, CYP2B6, CYP3A5, and CYP2D6 were poorly expressed in all segments. In the colon, the mean contribution to intestinal gene expression is: CYP2B6, 9%; CYP2C9, 0.8%; CYP2C19, 1.1%, CYP2D6, 2.4%; CYP3A4, 0.3%; CYP3A5, 3%. Sulfotransferases 1A (SULT1A), uridine 5′-diphospho-Glucuronosyltransferases 1A (UGT1A) and UGT2B7 were expressed in a similar longitudinal pattern: an increase was observed from duodenum to jejunum, followed by a substantial decline in the ileum and the colon [27].

Several DMEs have been reported to work in tandem with transporter proteins, including CYP3A4 and P-gp, UGTs and MRP2/3 as well as SULTs and BCRP [27]. Interestingly, the increase in P-gp expression from the proximal to the distal region of the small intestine coincides with a decrease in CYP3A4 expression [29,30]. Due to the synergistic interplay of CYP3A and P-gp on dual substrates and the long intestinal transit time, drugs can undergo extensive pre-systemic intestinal metabolism. Nevertheless, expression and protein levels do not always accurately reflect activity [30].

Drozdzik et al. reported a good correlation between the mRNA-protein levels of various transporters in jejunal tissue (i.e., P-gp and PEPT1, but not MRP3 and OATP2B1) [26]. Similarly, a study by Fritz et al. demonstrated good correlations between gene and protein expression for several DMEs (i.e., CYP3A4/5, CYP2C9/19, CYP2D6, UGT1A1/1A3 and UGT2B7) in small intestinal tissues, but not in hepatic tissue (i.e., CYP2E1, UGT1A3 and UGT2B7/15 and 46) [27]. However, overall gene expression data are not necessarily a good predictor for the encoded protein nor transporter capacity. Moreover, transporter capacity can be influenced by differences in surface area, blood flow, mode of action (pH—dependency of PEPT1 or ATP requirements of ABC transporters), and repeated cycles of metabolism [26]. Therefore, one should be careful to interpret mRNA/protein levels of transporters and enzymes in both in vivo and in vitro settings.

### 2.2. Mucus

The colonic epithelium is lined with a mucus layer, acting as a barrier to potential pathogens, but also to drug absorption [31]. Mucus is an aqueous matrix composed of water (95%), lipids, electrolytes and glycoproteins, with a thickness and composition that is region-dependent. The number of goblet cells increases from 4% in the duodenum to 16% in the distal colon [12,13], resulting in a thin and loosely structured mucus layer in the small intestine that allows an efficient drug and nutrient absorption (10 µm in the ileum) [32,33]. In contrast, the colonic mucus is organized as a thick two-layer system [34,35,36], and consists of a dense inner mucus layer (±100 µm in sigmoid human biopsies) which does not contain bacteria and is further remodelled by endogenous proteases into the thicker and more porous outer mucus layer (±300 µm) to allow a symbiotic relationship with the microbiome [14,15,27]. In contrast to the previous study that investigated mucus thickness solely in the sigmoid colon, Strugula et al. reported that the thickness of the adherent layer varies throughout the colon, ranging from 10–30 µm in the cecum to 30–85 µm in the rectal region. Possible discrepancies in mucus thickness between laboratories might be due to nonstandardized protocols [32]. The mucus properties, which include viscosity, mesh size, lipophilicity, and ionization state can influence drug diffusion and permeability, and require consideration when evaluating drug disposition.

Diffusion through the mucus is dependent on the physicochemical properties of the drugs and mucins. Mucins are highly glycosylated and negatively charged molecules at small intestinal and colonic pH, due to the low pKa (between 1 and 2.6) of the sialic acid at the terminal part of the oligosaccharide chain. MUC 2 is the predominant secreted mucin [16]. Desai et al. reported that several positively charged drugs (NAD, 5-hydroxy-L-tryptophan and 5-hydroxytryptamin) diffuse more slowly through porcine mucus than uncharged drugs [37], while anionic drugs can be repulsed by glycoproteins [38]. Hydrophilic and net-neutrally charged drugs are therefore favored for mucopenetration, as they will not form strong adhesive bonds with mucins. Behrens’ group compared the apparent permeability for a series of lipophilic drugs (including barbiturates and testosterone) between mucus-free Caco-2 cells and mucus producing HT29 cells, and reported a slower diffusion for drugs with logP > 1, e.g., up to 43% for testosterone [39]. Wikman-Lahred and colleagues came to a similar conclusion when using porcine intestinal mucus to investigate the diffusion of small molecules with logP values ranging from −3.1 to 3.3 [40]. The reduced diffusion of lipophilic drugs through mucus can be due to nonspecific binding to the lipidic nonglycosylated protein domains of the mucins [39,41]. Although hydrophilicity and charge-selectivity are important characteristics to achieve good mucus diffusion, hydrophobic drugs are more prone to partition into the lipid-dense cellular membranes.

Mucus is a randomly organized network of fibres, with a mesh size or diameter between 50 and 1800 nm in aqueous media. Common drugs with a size below 10 nm can freely diffuse through the mucus layer as the amount of steric interactions is limited. Even small capsid viruses (Norwalk virus, human papilloma virus, polio virus) can rapidly diffuse through human mucus [38]. Desai and colleagues reported a consistent retarding effect of mucus on the diffusion of macromolecules between 126 and 186,000 Da [42]. As such, size only has a substantial effect for very large drugs. However, since colonic conditions (limited and scattered free water pockets) are unfavorable for dissolving a drug which arrives in the colon in particulate form, mucus penetration may be restricted. The arrival of particles in the colon has recently been demonstrated by Lemmens and coworkers, who reported high solid fractions in the cecum for celecoxib and sulindac 7.5 h after oral intake [43,44]. Furthermore, size-dependent diffusion might be more restricted towards the distal part of the colon since the thickness of the viscous, gelatinelike mucus increases along the GI-tract. When a drug is subjected to suboptimal diffusion, either through interaction or size-filtering, the intestinal mucin turnover time can pose a prominent problem for drugs aimingto reach the epithelium. Mucus turnover and clearance varies from minutes to several hours and can prevent drug accumulation in tissue. The outermost mucus layer in the colon needs to be traversed especially quickly as it is also rapidly cleared [45,46]. Lastly, mucus contains digestive enzymes (including exopeptidases, such as carboxypeptidases and aminopeptidases) that can consequently affect the diffusion through the mucus layer [38]. To summarize, mucus penetration is favored by net-neutrality, an increase in hydrophilicity and decrease in size, and a good stability against mucus-located enzymes.

The role of mucus in drug permeation is increasingly explored, but suitable in vitro models are lacking. Intestinal cell line models such as Caco-2 are often used to assess permeability, but do not have goblet cells and thus lack a mucus layer. It is well known that the application of a mucus layer of porcine origin can circumvent this limitation [47], although the single and uniformly distributed layer does not accurately represent the colonic mucus that is organized as a thick two-layer system [36]. Furthermore, mucus from cell cultures possessing mucin producing cells can differ from native mucus in terms of mesh spacing or mucin expression pattern. For instance, the HT29 cell line mostly secretes MUC5AC instead of MUC2, the most dominantly secreted mucin in the colon under physiological conditions [48]. Mounting human colonic biopsies in Ussing chambers is a physiologically relevant model for studying the effect of mucus on drug permeation. It is noteworthy that the ex vivo conditions stimulate mucus production. Furthermore, this technique allows us to manually remove the mucus at the end of the experiment, enabling sophisticated studies investigating the effect of mucus on drug diffusion/accumulation. Human tissue samples are, however, short-lived (±4 h) and require invasive endoscopy, which is not feasible for routine applications in drug absorption studies. Recent progress has been made to culture intestinal crypts into gut organoids that represent a close approximation of the in vivo situation. For example, VanDussen’s group has reported that ileal and rectal organoids are capable of expressing a MUC2-rich and double layered mucus. The colonic organoids retained the complex and resistant mucus inner-layer after a “disruption” experiment, while the loose outer mucus layer was removed. However, the 36 µm thick mucus layer in the organoid monolayers does not accurately represent the thickness of the human mucus layer, which is approximately 400–600 µm [31,49]. The human colon chip that uses microfluidic culture technology has been successful in reproducing a physiologically relevant bilayered mucus structure with a total thickness of 500–600 µm [50]. Indeed, these dimensions are closer to the living human colon, although it should be noted that mucus thickness is region-dependent, even in the colon [27].

### 2.3. Microbiome

The human gut contains trillions of microbes, predominantly bacteria from hundreds of different species, with *Bacteroidetes* and *Firmicutes* being the most prevalent phyla, but also archaea, viruses and fungi. The microbiome exerts a crucial role in a range of physiological functions (e.g., food digestion, synthesis of essential vitamins, contributing to a functioning immune system, regulating intestinal functions, and others) and also produces organic compounds which fulfil an important physiological role (e.g., short chain fatty acids (SCFAs), phosphatidylcholine, branched-chain amino acids, vitamin K, secondary bile acids and neurotransmitters such as serotonin). Not only is the composition of the microbiome highly individual (influenced by, e.g., age, geographical location, diet, drugs, disease), the bacterial density and composition also varies along the GI tract. More than 400 species of bacteria are present, with high bacterial concentrations in the colon (10^11^ per gram content) compared to the proximal small intestine (10^4^ per gram) [51], which can be exploited for microbiome based colonic drug delivery (see Section 5.1.1.3). Moreover, most of the available studies have focused on the fecal, i.e., distal luminal microbiome, but this is different from the mucosa-adherent microbiome in the mucus layer or the lumen in the different regions of the GI tract which are more difficult to access [52,53]. For a detailed discussion of the microbiome diversity from multiple colonic mucosal sites and feces, and how it differs from the mucosa-adherent microbiome, we refer to studies by Eckburg [54] and Zoetendal [53], respectively.

After oral intake, many drugs can be modified by the gut microorganisms in the small and large intestine. Zimmerman et al. reported microbiome-induced alterations of two thirds of the 271 investigated drugs. This can result in their activation (e.g., sulfasalazine), inactivation (e.g., digoxin) or toxification (e.g., sorivudine) [55]. Furthermore, the microbiome can indirectly influence drug bioavailability by modulating the local environment by secretion of SCFAs like butyrate, or by altering the solubility and ionization state of drugs through fermentation products that result in pH fluctuations [35]. Likewise, dysbiosis in diseased states can be linked to an altered colonic transit, which impacts the extent of bacterial metabolism [56]. Lastly, an increase in the mucin-degrading bacterium *Ruminococcus gnavu* has been described, which can affect mucus diffusion [57]. For a more extensive study on human microbiome drug metabolism, we refer to [55].

## 3. Colonic Fluid Characteristics

### 3.1. Colonic Volume

Drug dissolution and release in the colon is influenced by a range of physiological variables, including pH, bile salts, pressure, buffer capacity, and fluid volume. The ascending, transverse and descending colon contain an average of 561 ± 321 mL of biomass, with 86% being water. Nevertheless, most of the water is bound to or contained inside the bacteria or biomass and not freely available to allow drug dissolution [58]. An MRI study showed a total free water volume in the colon of only 13 mL (range 1–44 mL) dispersed into small pockets [59]. 

However, the total free water volume is dependent on liquid and food intake. Murray’s group observed 11 ± 5 pockets of resting liquid with an average total volume of 2 ± 1 mL in the ascending colon in fasted state. Thirty minutes after the administration of a glass of water, 17 ± 7 separate liquid pockets were observed that amounted to a total volume of 7 ± 4 mL. This increase in the volume and number of pockets might be related to the gastrocolonic reflex, potentially transferring ileal contents into the ascending colon upon ingestion of water. An alternative explanation is that an initial fraction of the ingested liquid arrives in the colon [58]. Diakidou et al. compared the fluid volumes 5 h after ingestion of either a reference meal or 240 mL of water. They reported that fluid volumes were higher after food than after water intake in the distal ileum, cecum and the ascending colon (e.g., 22.3 ± 7.7 mL after water vs. 29.9 ± 10.8 mL after the reference meal in the ascending colon) and that liquid fractions were generally higher in the fasted than in the fed state [60]. Additionally, the fluid volume in the ascending colon is 5.9 and 4.2 times bigger compared to the ileum in the fasted and fed ileal condition, respectively [60]. This is in agreement with a regional analysis of Murray and colleagues, who demonstrated that free water pockets were found primarily in the ascending colon, although they noticed a very high interindividual variability [58]. This difference in volume might be due to a longer residence time in the ascending colon and to a change in bacterial content.

It is evident that a restricted colonic fluid volume can hamper drug dissolution and absorption. Tannergren’s group reported regional absorption data of 42 drugs demonstrating that most BCS I drugs (high solubility and high permeability) were well absorbed, implying a sufficient dissolution and uptake, either through convective or diffusive transport. However, some BCS II drugs (low solubility and high permeability) were associated with a lower colonic absorption compared to oral dosing (F_rel_ Colon: AUC_colon, local solution_/AUC_reference, oral administration_), despite being administered to the colon as solutions, which suggests in vivo precipitation [61]. A study by Lemmens et al. demonstrated that celecoxib and sulindac, two BCS II drugs, can be present in an undissolved state upon reaching the cecum by transit [43,44]. For both studies, it is presumably the viscous, limited, and scattered water contents that lead to the precipitation of already dissolved drug and obstruct further dissolution. This indicates that (i) low colonic fluid volume is an important criterium in early assessment of absorption potential for controlled release products (i.e., Tannergren and colleagues suggested the use of dose:solubility ratio of 50 mL for high solubility drugs) and (ii) that there is a need for a more relevant incubation method, e.g., suspensions or granules as opposed to solutions. Tannergren and colleagues showed that administering a suspension of dexloxiglumide (BCS II) to the colon resulted in a delayed maximal plasma concentration (T_max_) compared to a solution, which also indicates a slow dissolution rate. When the same comparative analysis was applied to BCS I drugs, a high relative colonic absorption was achieved, which is consistent with the higher solubility [61].

### 3.2. Colonic pH

Upon the arrival of carbohydrates in the cecum, the microbiome can transform undigested carbohydrates into SCFAs including acetic, propionic, and butyric acid. The fermentation of carbohydrates is accompanied by an intraluminal pH drop in the proximal colon (cecum or ascending colon). The pH drop averages between 1.5 (to pH 5.8–6.8) and 1.2 units (to pH 6.1–7.1) in the fasted and fed state, respectively, indicating that the food status only marginally affects luminal pH in the distal GI tract [62]. A gradual pH increase is described along the colon, reaching 6.1–7.5 in the rectum [63,64]. This pH rise along the colon is likely due to decreasing intraluminal SCFA concentrations [64]. Moreover, the colonic mucosal surface pH (7.1–7.5) [65] is reported to be higher than the luminal pH [64].

Besides prandial state, the effect of nonenvironmental factors such as gender and age on the pH profile has recently been reviewed by Broesder et al. [62]. The pH in the lower GI tract of fasted healthy children (age 8–14) differed only slightly from the pH observed in adults, while the effect of aging (age group 62–83) was inconclusive. Furthermore, gender showed no significant effect on the pH profile in the ileum and colon, although this was only investigated in the fasted state. 

Colonic drug delivery systems often rely on a pH trigger (among many other methods) to achieve a high and local disposition in the colon (see Section 5.1.1.1). The pH trigger is often the sharp local peak in the terminal ileum (>7.2), which initiates drug release and delivers the drug in close proximity to the cecum. The release should be achieved in the short ileal transit time window, as the relatively low cecal pH can impede further dissolution. However, interindividual pH variation can result in a suboptimal drug exposure, e.g., the maximum jejunal pH is reported to be 7.4, which initiates premature drug release when pH-dependent delivery systems are used. Furthermore, the pH in the diseased state (ulcerative colitis, Crohn’s disease, inflammatory bowel syndrome, and colorectal cancer) can deviate from a healthy status. Fallingborg and colleagues reported a pH as low as 2.3–3.4 in the proximal parts of three active UC patients [66]. For a more elaborate discussion on the GI pH profile in healthy and diseased state and how to apply it to in vitro testing we refer to [62].

### 3.3. Fluid Transport and Hydrodynamics

The smooth muscles of the gastric wall follow a cyclic iteration of three contractility phases, called the interdigestive migrating motor complex (MMC), which range from an absence of contractions (MMC phase 1) over irregular contraction patterns (MMC phase 2) to a short burst of contractions with maximal amplitude and frequency (MMC phase 3) that induce emptying of contents into the small bowel [67]. Segmental contractions start in the duodenum, become less spatiotemporally organized as activity propagates, and reach the terminal ileum in approximately 2 h [68]. This results in a net overall distal movement of chyme, which slows down substantially in the terminal ileum, resulting in a local temporary build up. The ileocolonic junction then regulates the passage to the cecum in a pulsatile contractive manner and is associated with propagating ileal contractions. Caloric intake can furthermore induce an ileocecal transfer. In total, a volume of 1–2 L reaches the cecum on a daily basis. The colon is characterized by rhythmic, phasic and tonic contractions that lead to both propulsion and mixing. Furthermore, high-amplitude propagating contractions induce mass movements and ultimately result in defecation [69]. The motility of the colon depends on many variables. For example, colonic transit is usually delayed during sleep, and motility increases significantly at awakening and with the intake of food. In addition, a gender effect was observed, with healthy women usually having a slower colonic transit (Maurer, 2016). Therefore, transit times are highly variable, e.g., Maurer et al. reported a colon arrival time of 3.25 to 8.20 h and a whole gut transit time of 10.01 to 59.39 h when using an IntelliCap^®^ system (an electronic oral drug delivery and monitoring device) [69]. The prolongation of the colonic residence time as compared to the small intestine (4 h) [70] in healthy subjects can be relevant for the disposition of colon-targeted delivery systems. For a more in-depth characterization of colonic transit, we refer to [69,71].

The release of drugs from colon-targeted formulations also indirectly depends on the peristaltic muscle contractions that control the hydrodynamics of colonic contents. As previously discussed, the colon is a particularly gas-dominated compartment with a low number of scattered water pockets. Schütt et al. studied the colonic fluid dynamics by computational techniques in three conditions: a completely and partially fluid filled model, and a partially filled colon with a gaseous phase. The group observed that the velocity and mixing profiles are condition-dependent: they are highest in the completely filled model, but similar for the partially fluid filled and gas-liquid model. The transit time of an undissolved tablet was highest in the completely filled model. These results show how colonic filling levels affect the prediction of the in vivo performance since it influences hydrodynamics, shear stress, mixing, and concentration. It is noteworthy that the study is more representative of the ascending colon where higher and less viscous contents are available, and transit time is less variable [72].

### 3.4. Buffer Capacity, Osmolality, Protein Content and Bile Acids

Human intestinal fluids contain bile salts, fatty acids, enzymes and other components that can impact the integrity of the mucosal barrier, as well as the dissolution and permeation rate of a drug. To this end, characterization of colonic fluids can result in a better understanding of drug absorption. Reppas et al. analyzed aspirated samples from the ileum, cecum and ascending colon, in both fed and fasted state. They reported that the pH and buffer capacity of cecal contents as compared to the ileum are lower and higher, respectively, which is the result of a higher concentration of total SCFA. Buffer capacity further increased towards the ascending colon. Prandial state did not influence SCFA levels in the ileal and cecal region but did in the ascending region. Furthermore, the osmolality of cecal and ascending colonic contents are lower compared to duodenal fluids; they are hypo-osmotic. While colonic protein contents are lower, total carbohydrate concentrations are higher in fed state conditions. Peptides and proteins are completely digested and the degradation products are largely absorbed along the proximal small intestine. This is not the case for carbohydrates, as the undigested fraction can reach the colon. The colonic concentrations of bile acids, which are largely reabsorbed in the distal ileum, vary between 70 µM and 500 µM in the fasted state. The composition is markedly different in the fed state. Consumption of a meal leads to a saturation of the metabolic capacity of the microbiome. Consequently, an increase in primary (cholic acid, chenodeoxycholic acid) over secondary (deoxycholic acid, lithocholic acid) bile acids is observed in the fed ascending colon. Free fatty acid concentrations are twice as high in the cecum and ascending colon compared to the ileum in both prandial states. An increased total concentration of phospholipids was observed from the distal ileum to cecum to ascending colon in the fasted state. In fed state conditions, the increase was only observed from the cecum to ascending colon. Cholesterol concentrations were not markedly different between the colonic regions, regardless of food intake [60,73,74].

The characterization of the composition of aspirated samples can improve our understanding of the performance of active pharmaceutical ingredients and has also been used to develop biorelevant media (see Section 6.2.1.3). Although the use of Fasted or Fed State Simulated Colonic Fluids (FaSSCoF and FeSSCoF) is still limited, other simulated fluids have proven useful to study drug behavior in in vitro models. The presence of bile acids in biorelevant media can positively influence the dissolution and solubility of lipophilic drugs. However, it has been shown that bile salts can alter the membrane fluidity of Caco-2 monolayers [75], and also affect ATP formation [76]. The effect of FaSSCoF and FeSSCoF on Caco-2 monolayer integrity or functionality has not been investigated so far. Similar to the reports on Fasted State Simulated Intestinal Fluid (FaSSIF) and Fed State Simulated Intestinal Fluid (FeSSIF), the components present in the biorelevant media may affect the barrier function. For instance, Ingels and colleagues showed how compounds in FaSSIF can modulate the function of efflux carriers. An inhibitory effect on P-gp was seen, especially for sodium taurocholate up to 3 mM [77]. Mixed micelles, present in biorelevant media, may affect the extent of drug absorption. Hydrophobic drugs have been reported to partition into these mixed micelles, thereby reducing the amount of free drug as driving force for permeating the Caco-2 layer [76,78]. The pH of the intestinal media will also affect the degree of drug ionization and permeation, i.e., weakly basic drugs such as propranolol and carvedilol can acquire a higher degree of ionization at pH 5 of FeSSIF, which could impair the permeation.

It is noteworthy that biorelevant media are still an approximation of the complex in vivo fluid composition. For example, the total bile salt concentration, phospholipid content, pH, osmolality and buffer capacity differ between human and simulated intestinal fluids, and therefore might not accurately predict the in vivo anticipated food effect on drug absorption. Furthermore, biorelevant media are not always compatible with intestinal models, as high and toxic concentrations of bile acids/lipids can solubilize the cells and affect integrity. However, the detrimental effect of biorelevant components on the integrity of Caco-2 cells could be minimized, as observed for simulated small intestinal fluids, by reducing the concentration of bile salts [79] or increasing the concentration of phosphatidylcholines [80]. Intestinal Caco-2 monolayers are also unable to produce mucus, which provides protection against endogenous compounds in the intestine. This can be circumvented by applying an artificial porcine mucus layer on top of the monolayer [81].

## 4. Pathophysiology

Inflammatory bowel disease (IBD) is a disease characterized by chronic inflammation of the GI tract and manifests clinically mainly in two disease forms: Crohn’s disease (CD) and Ulcerative colitis (UC). Although the etiology remains unknown, it is clear today that IBD is the result of an overactive immune system involving genetic, environmental and microbial factors. CD is characterized by transmural inflammation that can occur discontinuously over the entire GI tract (small and large intestine), while lesions in UC are restricted to the mucosa and submucosa of the colon only [82]. IBD, especially in case of ongoing inflammation, is linked to an abnormal gut barrier [83] that subsequently also affects drug absorption. For example, Johansson and colleagues showed that colonic mucus properties can be modulated in CD and UC animal models and sigmoid colon tissue of active UC patients, thereby also allowing bacteria to penetrate the inner mucus layer, and come into close contact with the epithelium [31]. Furthermore, microbial dysbiosis in IBD, which is characterized by reduced diversity and temporal stability, and a decrease in *Firmicutes* and an increase in *Proteobacteria* at phylogenetic level [84], can dysregulate barrier function. The depletion of families belonging to *Firmicutes* can result in a functional disturbance, such as lower butyrate-producing capacity of the IBD microbiome which subsequently can reduce epithelial barrier integrity and promote inflammation [85]. A recent publication reported that increased intestinal permeability can precede the disease onset by as many as three years [86]. UC and CD are also linked to a shortened transit time (on average 24 h in UC and CD and 52 h in healthy volunteers), which can be detrimental for formulations with slow release or dissolution of the drug. Moreover, a shortened transit can potentially alter the local pH, since the pH can increase when the microbiome produces less SCFA due to time constraints, although a lack of SCFA can cause bacteria to produce lactic acid, which can decrease pH [64]. The GI pH profile in patients with CD and colorectal cancer has not been shown to deviate from healthy volunteers, while distinguishable pH values have been demonstrated for UC patients. For patients with irritable bowel syndrome, the mean ileal pH was 7.7, which falls within the healthy pH range (7.2–7.7), but the mean cecal pH was 5.1 and is thus lower than the minimum healthy ileal pH (5.7) [62]. Specifically, the disposition of colon-targeted formulations that require a pH trigger can be suboptimal because of deviating pH and transit times. As such, numerous physiological variables (pH, microbiome composition, transit time, and others) are affected by colonic diseases, which subsequently also alter drug absorption.

## 5. Formulation Strategies

### 5.1. Oral Colon-Targeted Drug Delivery Systems

Colon-targeted drug delivery systems have been explored increasingly over the past few decades for local treatment. Formulations are designed to undergo little to no degradation during their transit through the gastric and small intestinal environment, thereby preventing premature release and decreasing the risk for adverse effects. They are usually engineered to be triggered by colonic conditions for which various strategies are currently used, e.g., pH-, enzymatic degradation- and time-dependent release forms. These systems mainly focus on a single mechanism of drug release. Single strategies can be combined (e.g., multimatrix system) and hold the potential to overcome disadvantages of single conventional release systems: e.g., sustained release after a specific pH threshold will overcome the possible risk of dose dumping and premature release of a pH-dependent release system. Other approaches might be interesting topics for future research for colon targeting (e.g., nanodelivery systems). It has to be kept in mind that reabsorption of water causes colonic contents to be highly viscous, which may limit site-specific delivery [87,88].

Below, we briefly discuss (i) formulations that are currently used for colonic release and (ii) novel, investigational, as-yet unvalidated strategies for colon targeting (Table 1).

#### 5.1.1. Currently Available Colon-targeted Drug Delivery Systems

##### 5.1.1.1. pH-Dependent Release

Drug products can be coated with pH sensitive polymers or embedded into pH sensitive matrices and require a pH trigger to achieve local drug delivery. The polymers used include cellulose-, acrylic acid derivatives, and copolymers of methyl methacrylate (Eudragit^®^) with threshold pH values varying between 4.5 and 7.0. The selection is based on the region specific pH to initiate drug release, and is thereby supposed to prevent premature release [82], which can best be achieved with a polymer with a narrow pH release range. As mentioned in Section 3.2, Broesder et al. reported a slightly higher pH value (7.2–7.7) in the terminal ileum resulting in an ideal pH threshold for ileocolonic drug delivery. This slightly higher pH in the ileum is followed by a pH drop in the cecum.

While pH-dependent mesalazine formulations with Eudragit-L coating disintegrate at pH ≥ 6, thus releasing the drug in the mid to distal ileum and colon (e.g., Salofalk^®^, Mesasal^®^ and Claversal^®^), Asacol^®^ is manufactured with Eudragit-S coating, which disintegrates at pH ≥ 7, thereby releasing mesalazine in the terminal ileum and colon. [89]. As various pH-dependent drug delivery systems rely on this slightly higher pH for ileocolonic release, it is important to assess the performance (or dissolution) of such systems through in vitro dissolution methods simulating the GI pH profile (see Section 6.2.1). As previously mentioned, colonic diseases may alter the pH profile. It is therefore crucial to evaluate the drug release in patients and have in vitro tools available mimicking the diseased state [62].

##### 5.1.1.2. Time-Dependent Release

Time-dependent release systems aim to deliver the drug to specific regions after a predefined time window. These formulations suffer from variability due to the interindividual differences in GI transit times [90]. The advantage of this release mechanism is its independence from interindividual pH variability and its gradual distribution throughout the GI tract. However, the latter one is also considered as a disadvantage as the continuous release throughout the GI tract results in increased systemic side effects, due to higher systemic absorption, and reduced drug availability at the disease site, the colon [91]. Pentasa^®^ is an example of an ethylcellulose-coated tablet which results in a gradual release of mesalazine from the duodenum to the rectum [89]. Other systems for time-dependent release have been developed and rely on swelling, osmosis or a combination of both [92].

##### 5.1.1.3. MultiMatrix System (MMX)

Mezavant^®^ is a mesalazine formulation based on a multimatrix system, in which mesalazine is incorporated in a lipophilic matrix which is dispersed in a hydrophilic matrix. The tablet is enterically coated to neutralize an effect of the variable residence time in the stomach. The tablet disintegrates at pH ≥ 7 in the terminal ileum. After disintegration, swelling of the hydrophilic matrix is initiated. The viscous gel that is then formed results in the slow diffusion of mesalazine so as to ensure release of the drug throughout the total length of the colon [93].

A multimatrix system has also been developed for budesonide [94]. The multimatrix system combines the pH threshold at the ileocolonic junction and a diffusion-based release mechanism. Combining systems holds the potential to overcome disadvantages of release based on a single system.

##### 5.1.1.4. Bacterial Degradation (Enzymatic)

Formulations and prodrugs intended for colonic delivery often make use of bacterial degradation by colonic enzymes present in the microbiome such as glycosidase, xylosidase [95], galactosidase [96], nitroreductase [97] and azoreductase [98] to cleave glycosidic bonds, α- or β-glycosidic linkages (chitosan), β-galactosides into monosaccharides (guar gum), to reduce nitro substituents on aromatic rings and to cleave azo bonds, respectively. The best known prodrug designed for colonic drug delivery is sulfasalazine. This prodrug passes the upper GI tract intact due to extensive efflux, and undergoes azoreductase-mediated biotransformation in the colon [99]. Although no polysaccharide-based delivery systems are commercially available, the utility of polysaccharide matrices is promising as degradation can be exclusively initiated by the colonic microbiome [100] and is not affected directly by colonic variables such as pH and pressure phenomena. However, dysbiosis, which is associated with a diseased state (e.g., IBD patients), can affect the degradation rate of polysaccharide matrices, such as locust bean gum, dextrans, chondroitin sulphate, cyclodextrins, and others [101].

#### 5.1.2. Investigational Colon-targeted Drug Delivery Systems Relying on (patho)physiology

##### 5.1.2.1. Size-Dependent Nanodelivery Systems

Size reduction of formulations to a nanoscale (i.e., nanoparticles) has been linked to an improved drug exposure to inflamed colonic tissue [102]. Nanoparticles may improve the treatment of IBD patients who often suffer from diarrhea and thus a fast colonic transit (‘streaming’). It has been described by Beloqui and coworkers that, in mice with induced colitis, budesonide-loaded nanostructured lipid carriers showed a sustained residence time in the colon, even when mice were subjected to diarrhea [103]. This suggests that it is possible to avoid rapid dosage form elimination by reducing the particle size. Extrapolation to humans remains to be explored. Nanosized drug carriers may also result in increased penetration in the mucus layer [104,105,106]. In addition to the reduced size, the type of nanomaterial and shape can also be modified depending on the clinical applications. Since we focus on the potential of the different formulation strategies, rather than the technology, we refer to a recent review of Mitchell et al. who discussed nanoparticle designs for precision applications [107].

##### 5.1.2.2. Positively Charged Nanodelivery Systems—Mucoadhesives

Positively charged nanodelivery systems can be designed to adhere to the anionic mucins of inflamed tissue resulting in prolonged local drug release [108]. Subsequently, diffusion of the drug depends on its physicochemical properties and the properties of the mucins (see Section 2.2). Mucoadhesion can especially be a promising strategy to target Crohn’s disease, as inflamed tissue has been linked to a higher level of mucus production [109,110]. Local delivery of these systems may prevent premature loss of mucoadhesive drug delivery systems due to binding to mucins in the upper GI tract. Many examples of positively charged nanocarriers have been reported in the literature. One example investigated by Niebel et al. is clodronate-loaded nanoparticles which were functionalized by the cationic ligand polymethacrylate [111]. For a more detailed overview of positively charged nanodelivery systems, we refer to the recent reviews of Hua et al. and Lu et al. who discussed oral nanodelivery systems for colon targeting [105,112].

##### 5.1.2.3. Pressure-Controlled Release

The phasic and tonic contractions in the colon, combined with the viscous colonic contents result in a high mechanical stress relative to the small intestine. This underlies the development of pressure sensitive formulations so that disintegration is only initiated in the colon [113]. Takaya and coworkers developed pressure-controlled colon delivery capsules using the insoluble polymer ethyl cellulose [114]. The pressure in the colon results in the disintegration of the insoluble polymer capsule, which initiates drug release. Whereas the amount of water is the most important factor for osmotic-controlled release (see below), the thickness of the ethyl cellulose membrane mainly determines disintegration of pressure-controlled release systems [113]. However, the low availability of colonic fluids hampers drug dissolution. To circumvent this issue, the drug can be included as a solution in these pressure-controlled ethyl cellulose capsules [114]. Although promising, pressure sensitive formulations are not commercially available.

##### 5.1.2.4. Osmotic-Controlled Release

Another possible approach to achieve sustained colonic delivery is the osmotic controlled release systems (OROS). The OROS system contains an osmotic push compartment and a drug reservoir surrounded by a semipermeable membrane. When water enters the osmotic push compartment, the drug is forced out the tablet through a laser drilled hole [115]. To prevent immediate drug release after contact with luminal contents, and to ensure colonic drug delivery, the semipermeable membrane of the OROS system can be coated with a polymer which dissolves at the slightly higher pH at the end of the ileum (pH > 7). In this manner, drug release is only initiated when the OROS system reaches the colon [116]. Although this technology holds potential, it has not been developed for the treatment of bowel diseases yet [117]. Besides, the low availability of water in the colon may limit the use of these systems for colon-targeted drug delivery (see Section 3.1).

### 5.2. Rectal Drug Delivery Systems

Although rectal delivery systems show favorable efficacy and safety profiles due to local administration, these delivery systems are less frequently considered as first line treatments in IBD compared to oral therapies due to low compliance, leakage, bloating and local discomfort after rectal administration. Nevertheless, different topical formulations (e.g., suppositories, foams, gels, and enemas) have been developed, and used as monotherapy or in combination with oral therapy for the treatment of distal forms of UC (e.g., ulcerative proctitis, ulcerative proctosigmoiditis, left-sided colitis). The benefit of these therapies includes direct delivery to the inflammatory region, rapid response, once-daily dosing, and low systemic drug exposure [118]. Regarding the site of action of the different topic formulations, suppositories disperse in the rectum, foam reaches for the sigmoid and descending colon, and enemas can reach the splenic flexure [119]. Rectal drug delivery systems thus target only the distal parts of the colon.

## 6. Colonic Drug Disposition and Evaluation Tools

### 6.1. Human Colonic Drug Disposition

Drug release, dissolution, precipitation, permeability and metabolism are the main processes determining local concentrations and bioavailability. As discussed previously (see Section 3), drug release and dissolution in the colon are influenced by a range of physiological variables, including pH, bile salts, pressure, buffer species, and fluid volume. The reported limited colonic fluid volume and the peristaltic muscle contractions that control the hydrodynamics of the colonic contents are important considerations in the early assessment of the colonic disposition of colon-targeted drugs (see Section 3.1 and Section 3.3). Furthermore, the microbiome can indirectly influence drug disposition by modulating the intestinal barrier function by the production of SCFAs like butyrate, or by altering the solubility and ionization state of drugs through fermentation products that result in pH fluctuations (see Section 2.3). In addition, disease state conditions should be included in dissolution studies as well (see Section 4). It is obvious that colonic drug disposition is the result of various simultaneously ongoing processes which are difficult to capture in vitro. It is also noteworthy that in vivo assessment of colonic drug disposition is extremely challenging.

Different techniques can be used to assess in vivo colonic drug disposition, including gamma-scintigraphy. Edsbäcker et al. [120] used gamma-scintigraphy to assess budesonide release from the orally taken controlled release capsule Entocort^®^ in healthy volunteers and CD patients. Budesonide was deuterium-labelled, and its release was followed by a gamma camera while blood samples were taken. Gamma-scintigraphy provides information about the drug release from formulations in different regions. From these release data, predictions concerning drug dissolution and possible uptake into the colonic regions can be made. Blood concentration data can be used to estimate drug dissolution by deconvolution based on predictive mathematical models [121].

Several research groups rely on fecal samples to estimate colonic drug release and concentrations, e.g., Yu et al. determined mesalazine concentrations in fecal contents after the administration of locally acting formulations [122].

However, the approaches mentioned so far only give an indirect idea of drug and formulation behavior in the colon. It would be more relevant to be able to determine local in vivo concentrations in the colon. Unfortunately, the collection of time-dependent colonic contents is not possible due its high viscosity. Single time point collection of the colonic contents during endoscopy has been shown to be feasible and these aspirates can be characterized in terms of pH, lipids, bile salts, buffer capacity, etc. [60,74] and used to facilitate the development of biorelevant media [123]. Collected colonic fluids can also be used to measure drug solubility post-collection [60,74,123].

**Figure 1:**
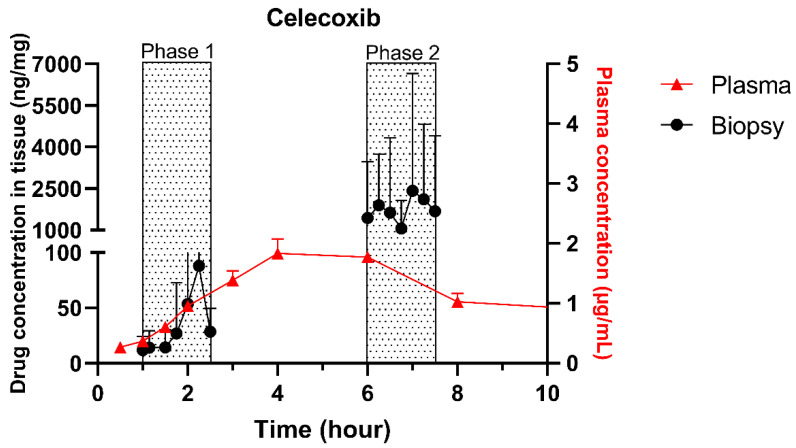
Concentration–time profile of celecoxib in plasma samples (▲) and cecal biopsies (●), collected from healthy volunteers after oral intake of one tablet of Celebrex (200 mg celecoxib) with 240 mL of water under fasted conditions. Adopted with permission from [43], Elsevier, 2020.

The single collection of colonic contents after oral drug intake has recently been combined with the time-dependent collection of biopsies as an indirect measure of luminal colonic drug concentrations. More specifically, several in vivo studies were conducted to clarify the disposition of celecoxib and sulindac at the level of the cecum. By collecting the colonic contents, the study showed that an undissolved fraction of NSAIDs is able to reach the colon by transit 7.5 h after oral drug intake, despite not being dosed as a colon-targeted formulation. The collection of biopsies revealed subsequent gut-driven uptake, which could eventually improve the anticarcinogenic effect of NSAIDs (Figure 1) [43].

It is obvious that the challenges associated with in vivo sampling studies, including their invasive nature, the colonic cleansing procedures, and sedating drugs altering the local physiology, the labour intensive sampling of colonic contents and biopsies, and the limited colonic contents call for viable alternatives to explore colonic drug disposition.

During drug development, in vitro release and dissolution setups continue to be essential tools, especially given the wide range of physiological variables (including pH, bile salts, pressure, buffer species, and fluid volume) that can affect colonic drug disposition. The compendial methods commonly used for the evaluation of immediate release and modified release systems such as United States Pharmacopeia (USP) dissolution apparatus II (paddle method), USP apparatus III (reciprocating cylinder), and USP apparatus IV (flow through method), and modifications thereof, have also been implemented in the evaluation of drug release in the lower intestine. For instance, Andreas et al. [124] used the USP III and USP IV methods for the evaluation of the release behavior of delayed and extended release mesalazine formulations as well as the evaluation of food intake on drug release. Additional examples of evaluation methods of drug products with release in the lower intestine can be found in Section 6.2.1.3. For a more extensive discussion we refer to other reviews [125,126,127]. The media used in these dissolution studies are obviously also very important to create relevant conditions. Various media used for the evaluation of colonic drug release, including simple buffer systems, biorelevant media, and media supplemented with rodent cecal contents as well as human fecal slurries, are discussed below.

### 6.2. In Vitro Evaluation Tools

#### 6.2.1. Dissolution

To evaluate colonic release and dissolution, different in vitro setups and models have been developed. In this review, we mainly focus on the media used in in vitro dissolution systems. For an overview of the in vitro models, we refer to other reviews [73,127,128]. Physiological buffers to which enzymes, relevant intestinal components (e.g., bile salts and lecithin), rat cecal contents and human fecal contents have been added for in vitro evaluation of colonic drug delivery systems will be further discussed.

##### 6.2.1.1. Physiological Buffers

As pH, ionic strength and buffer capacity affect drug release, physiological buffers are commonly used for dissolution and release studies. USP buffers (e.g., hydrochloric acid, phosphate, acetate and citrate buffers) have been widely used and shown to be valuable for the assessment of drug release and dissolution. In addition to the USP buffers, bicarbonate media have been implemented as dissolution media to better simulate small intestinal luminal fluid. For instance, Fadda et al. [129] used bicarbonate media in the USP II dissolution apparatus for the evaluation of formulations designed for ileo-colonic delivery. They showed that the use of bicarbonate buffer resulted in improved predictions of the in vivo behavior of mesalazine formulations, compared to compendial buffer.

The high volumes often used in the dissolution setups remain questionable for predicting the dissolution behavior of colon-targeted drug delivery systems, as high volumes may result in an overestimation of the dissolution. Downsizing the in vitro dissolution setups may result in a better approximation (e.g., minipaddle apparatus, see Section 6.2.1.3).

The composition of colonic fluids also deviates from simple aqueous buffer systems, necessitating the modulation of the media used. For instance, the absence of bile salts (e.g., responsible for an increased solubility) may result in an underestimation of the dissolution. In addition, these buffers lack the enzymes responsible for the degradation of controlled release formulations (see Section 5.1.1.3). By adding e.g., enzymes or relevant intestinal components, some of those complex factors for degradation can be addressed in a simple setup, thereby dissecting the complexity of colonic contents into individual defined contributions.

##### 6.2.1.2. Inclusion of Colonic Enzymes

As previously described (see Section 5.1.1.4), the release from formulations which depend on bacterial degradation is rendered by the intestinal microbiome. The enzymes responsible for degradation can be included in physiological buffers. They are considered easy to handle and inexpensive. Although a defined mixture of enzymes does not compare to the diversity of bacterial enzymes in the colon, Siew et al. [130] demonstrated a good correlation between selected amylases and fecal dissolution systems (see Section 6.2.1.4) when investigating amylose formulations. Wahlgren et al. [127] reported on the use of other enzymes in dissolution studies, e.g., azoreductase, galactomannanase, α-galactosidase, β-glucosidase, pectinase, dextranase, and inulase. The inclusion of rat cecal or human fecal contents can be considered as an alternative to defined single or combinations of enzymes.

##### 6.2.1.3. Inclusion of Relevant Intestinal Components

Since plain aqueous physiological buffers are not ideal for the prediction of the in vivo release of poorly soluble drugs (BCS class 2 and 4), biorelevant media have been developed, containing relevant bile components and reflecting the physiological pH, osmolality, and surface tension. For the development of biorelevant media representative of the lower intestine, Reppas et al. [74] analyzed aspirated samples from the ileum, cecum and ascending colon, in both fed and fasted state (see Section 3.4). The composition of these media simulating the distal ileum (simulating intestinal fluid, SIF_ileum_), ascending colon in the fasted state (fasted state simulated colonic fluid, FaSSCoF) and in the fed state (fed state simulated colonic fluid, FeSSCoF) are listed in Table 2. All these media differ in pH, buffer capacity, osmolality and bile salts. Vertzoni et al. [123] used these media (FaSSCoF and FeSSCoF) to determine the solubility of poorly soluble drugs (ketoconazole, danazol and felodipine) in comparison with the solubility in plain buffers. Georgaka et al. and Markopoulos et al. implemented the biorelevant media into dissolution tools. In both studies [131,132], dissolution in the distal ileum and the proximal colon (two-stage) was evaluated in fasted and fed state conditions. Briefly, in the first stage of the dissolution setup, SIF_ileum_ is added, and after a certain time (0–2 h) FaSSCoF or FeSSCoF is created (second stage; 2–6 h); Georgaka et al. made use of SIF_ileum_-V2 and FeSSCoF-V2. Georgaka et al. [131] described a two-stage single-compartment model making use of the minipaddle apparatus; this two-stage in vitro evaluation simulates dissolution in the distal ileum and proximal colon, thus creating the possibility to evaluate low solubility drugs and pH-dependent release formulations in two different media differing in bile salt composition and pH. Markopoulos et al. [132] compared the minipaddle apparatus (with the paddle rotating at 100 rpm) to the USP apparatus IV (flow-through apparatus), to explore the effect of differences in mechanical stress on the dosage form by agitation by a different type/intensity of fluid convection. This approach has, for instance, been used to evaluate the dissolution of Asacol^®^ tablets (pH-dependent release colon-targeted drug delivery system of mesalazine).

##### 6.2.1.4. Inclusion of Intestinal Contents (Rat Cecal and Fecal Slurries)

As previously described (see Section 5.1.1.3), formulations and prodrugs intended for colonic delivery often make use of bacterial enzyme-mediated degradation. To explore this degradation, the inclusion of colonic enzymes can be considered. Alternative approaches have been developed to better represent the colonic environment by using rat cecal slurries, human fecal slurries and multistage culture systems (see Section 6.2.4.1 and Section 6.2.4.2).

Rat cecal contents have been widely utilized in drug release and dissolution testing. Due to the anaerobic nature of the cecum, rat cecal contents are isolated and immediately diluted and transferred under anaerobic conditions to sealed glass vials. Its relevance has been reported by Hawksworth et al. [133] as similar amounts of the bacteria *Bacteroides* and *Bifidobacteria* were observed in human colonic contents as in rat cecum. Another advantage is that rat cecal contents are readily available and donor-dependent variability can be decreased by standardized housing conditions. The usefulness of this approach has been demonstrated in various studies: the presence of cecal contents resulted in increased drug release from a guar gum matrix (indomethacin [134]), calcium pectinate tablets (indomethacin [135], 5-fluorouracil [136]), chitosan tablets (Ibuprofen [137]), calcium pectinate microbeads (satranidazole [138]), and enteric coated guar gum microspheres (ornidazole [139]). By incubation in a rat cecal contents containing-medium, Yassin et al. [140] demonstrated the susceptibility of chitosan coated 5-fluorouracil tablets for the enzymatic degradation by colonic enzymes. The in vivo selectivity of the system resulting in colon targeting was confirmed by X-ray imaging using beagle dogs.

Although the use of rat cecal contents has been valuable to explore enzyme-mediated colon drug delivery systems, the results obtained with rat cecal contents containing media may not always be extrapolated to humans due to differences in the microbiome (e.g., *lactobacilli* in humans are present in a lower proportion compared to rodents).

Alternatively, feces collected from humans can be used to prepare slurries in which the performance of colon specific drug delivery can be explored. This approach has the advantage that samples from healthy volunteers as well as fecal samples from patients can be used. The anaerobic conditions require that the experiments are to be performed in an anaerobic workstation. A disadvantage of this approach is the fact that the microbiome and composition in the lower colon differs from the composition in the upper colon. A large variation in the composition of fecal samples from different individuals also needs to be considered [127]. Different research groups have shown the applicability of fecal slurries for assessing the performance of colonic drug delivery systems that depend on the presence of the colonic microbiota. For instance, Milojevic et al. [141] tested the release of mesalazine from pellets coated with amylose/ethylcellulose while using human fecal slurries prepared from feces of healthy volunteers. It was observed that mesalazine was completely released within 6h in the fermenter whereas negligible release was observed within 12h in simple buffer systems. The release occurred because of the degradation of amylose rather than the physical destruction of the coating. Similar experiments with comparable outcomes have been performed by McConnel et al. [142] and Salunkhe et al. [143]. Furthermore, the release of mesalazine from pellets coated with starch derivatives (Nutriose) was evaluated in the presence of fecal contents from IBD patients [144,145]. They observed that drug release significantly increased in a time-controlled manner upon contact with fecal contents, but not in the media that lacked a microbiome. It should be considered that the microbiome differs in IBD patients as compared to healthy humans and that this may have an impact on the performance of colon-targeted formulations (see Section 4). As such, using fecal contents can be useful to evaluate microbiome based drug release from different donor groups (e.g., healthy and diseased), and is therefore implemented in various multicompartment models such as the three-stage compound continuous culture system [146,147], the Netherlands Organization for Applied Scientific Research (TNO) Gastro-Intestinal Model (TIM) and the Simulator of the Human Intestinal Microbial Ecosystem (SHIME^®^) (see Section 6.2.4). Nevertheless, the complexity of the models limits throughput, and donor availability (having access to the right donor population depending on scientific question) as well as heterogeneity of the microbiome, remain challenges for study design and data interpretation.

#### 6.2.2. Drug Degradation

In addition to prodrug activation or degradation of colon specific drug delivery systems, the colonic microbiome may also contribute to drug degradation, thereby decreasing the amount available for colonic absorption [61]. Fecal material can be used to assess ex vivo drug degradation [148]. Tannergren et al. [149] showed the in vivo relevance of using fecal homogenates from healthy human volunteers, dogs and rats in stability studies, using almokalant, budesonide and ximelagatran as model compounds. Metoprolol was used as negative control as it does not undergo bacterial degradation. The three model compounds underwent colonic degradation; the in vivo relevance was demonstrated by a correlation coefficient (R^2^) of 0.90, which was obtained between the percentage of drug remaining after 60 min of in vitro incubation and the fraction absorbed (F_a_) after colonic drug administration in humans. As discussed before, fecal contents are not truly representative of the proximal colon. To circumvent this issue, Vertzoni et al. [150] performed stability studies using contents collected from the ascending colon. The mean half-lives of metronidazole and olsalazine were determined using ascending colon contents and fecal contents collected from healthy adults [60,151]. These experiments revealed that degradation in ascending colon contents was significantly lower compared to degradation in fecal material. In view of the difficulties associated with the collection of colonic contents, the use of fecal material remains a valuable alternative.

As mentioned previously, the diseased state may also affect drug degradation. For instance, Fabia et al. [152] and Pathmakanthan et al. [153] reported a significant decrease in anaerobic bacteria in UC patients. Together with the fact that Carrette et al. [154] reported decreased azoreductase activity in CD patients, it is expected that drugs such as metronidazole and olsalazine are more stable in IBD patients than in healthy subjects.

#### 6.2.3. Drug Absorption and Stability

For drug evaluation, intestinal setups need to represent the biochemical (metabolism, active transport mechanisms) and physical (surface area, cell-types, mucus production, unstirred water layer) characteristics of the GI tract to enable an accurate prediction of bioaccessibility (a drug that is released from a matrix and becomes available for absorption), permeability, and stability. We will discuss different traditional as well as recently emerged novel intestinal in vitro tools and compare them concerning suitability to evaluate colonic drug behavior. Intestinal in vitro tools can be categorized into systems derived from (i) immortalized cells (“gold standard for permeation studies”; Caco-2), (ii) ex vivo (Ussing chamber) and (iii) primary systems (primary enterocytes, Cryopreserved human intestinal mucosa CHIM^TM^)), (iv) stem cell-derived models (intestinal organoids, scaffold-based models, and Gut-on-a-Chip), and (v) in vitro tools to study the role of the microbiome (TIM and SHIME^®^). Traditionally available intestinal in vitro systems have successfully been used in the past to identify high and low permeable drugs (Caco-2) and CYP3A4-dependent metabolism (intestinal subcellular fraction, intS9). However, accurately assessing transporter-dependent efflux or uptake can sometimes be challenging with the Caco-2 system due to its different expression patterns compared to human intestinal tissue [155]. To accurately assess intestinal metabolism intestinal microsomes or intS9 are traditionally used, containing only microsomal or microsomal and cytosolic Phase I (CYP450) and II enzymes (especially UGT), respectively, but lacking several mitochondrial (monoaminoxidase) enzymes, overall often not capturing nonCYP3A metabolism well [156]. Ex vivo systems like the Ussing chamber and primary enterocyte systems resemble intestinal physiology but suffer from limited availability, and the absence of expansion capacity. The timeframe within which tissues and cells can be used is a limiting factor as well. Stem cell-derived systems have originally been used for disease modelling and regeneration studies. More recently, those systems have also been utilized to characterize drug disposition and safety [2,157,158,159]. Those systems emulate intestinal physiology and are designed for longer-term culturing. Several of the stem-cell derived models discussed below are currently only available (or described in the literature) as small intestinal models and therefore differ from the scope of the review. However, since the methodologies and platforms can likewise be applied to the colon and colonic models are often either in development, already available (but data concerning DMEs, transporters and functional characterization are often still limited or not available in the public domain yet) or can be foreseen, we include here a summary of the currently available small and large intestinal models.

##### 6.2.3.1. Available as Colonic Models

*Traditional Immortalized Cell-Lines: Caco-2*

**Figure 2:**
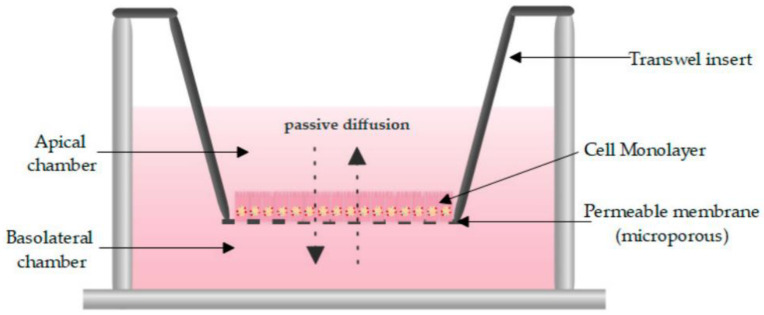
Schematic overview of a Caco-2 monolayer on a transwell insert. Adopted from [164], MDPI, 2019.

One of the most routinely used in vitro set-ups for the prediction of human drug absorption is the Caco-2 cell culture model. The cell line, cultured as a monolayer, originates from colorectal carcinoma cells that spontaneously differentiate into columnar enterocytes, after having reached confluency on a porous membrane (Figure 2) [1]. After three weeks (21–28 days), the cells resemble the human epithelium, which in terms of morphology (polarity, tight junctions, brush borders with microvilli) and functionality (passive and active transport mechanisms, limited metabolic capacity) is in many ways more representative of the small intestine than the colon. Furthermore, the lack of mucus producing goblet cells makes the monolayer more similar to the small intestine than to the colon. The system has shown strong correlations between F_a_ and the apparent permeability for a series of passive diffusion drugs with heterogeneous structures [160,161], allows classification into completely absorbed and poorly absorbed drugs [162], and can do so in a high throughput and reproducible manner. Tannergren et al. were able to establish a sigmoidal relationship (R^2^ = 0.74) between the apparent permeability coefficients (P_*app*_) of 18 drugs and their corresponding colonic absorption in humans, expressed as F_rel_ (see Section 3.1) [61], likely due to its colonic origin. This validates the value of using Caco-2 in the early assessment of colonic absorption potential for controlled release drugs. Nevertheless, there are several shortcomings: Caco-2 cell monolayers have a physiologically high tightness (a tight junction pore radius that is smaller than native tissue) [163], the enterocytes lack the main intestinal metabolising enzymes (i.e., CYP3A4), and drug transporter expression levels are not always reflective of the in vivo levels, which partially explains why a poor correlation exists for drugs that are transported via carrier-mediated mechanisms [160]. Furthermore, the thickness of the unstirred water layer can deviate from the in vivo state, and there is no mucus lining.

Although the motility and the low water content in the colon minimize the thickness of the in vivo unstirred water layer (<100 µm), it can limit the permeation of lipophilic drugs. The thickness of the unstirred water layer can be up to 2000 µm in the Caco-2 system [165], but stirring devices that simulate intestinal motility can reduce it. A second in vivo barrier is the presence of a thick colonic mucus layer that can affect drug permeation and the degree of interaction between drugs and epithelium, which has been discussed previously. To circumvent the lack of mucus in the Caco-2 model, several strategies can be employed. Unpurified and porcine mucin resemble human mucins in molecular weight, structure and mesh size [166]. This mucin solution can be pipetted on top of the Caco-2 monolayer [81]. Stappaerts et al. applied this approach and showed how mucus can indeed limit diffusion and how this is drug-dependent: the more lipophilic heptylparaben diffused more slowly in the presence of mucus while the effect was negligible for methylparaben, which is only slightly lipohilic [167]. Coculture systems are also an option for exploring the effect of mucus as a transport barrier. For instance, HT29-MTX (human colorectal adenocarcinoma cell line differentiated into mature goblet cells using methotrexate) allows mucus production and also mimics the physiology of the human intestine more closely. Interestingly, including HT29-MTX did not result in a better correlation with human F_a_ for passive diffusing drugs [168].

However, transporter and drug metabolizing enzyme levels are not always accurately captured by the Caco-2 model. Although DMEs such as UGTs are present, several metabolizing enzymes are missing or not expressed at physiologically relevant levels. For example, carboxylesterases-1 (CES) is overexpressed while CES-2 is underexpressed in Caco-2 cells compared to the small intestine. In particular the lack of CYP3A4, which accounts for 80% of total CYP in human small intestine, is disadvantageous when the drug of interest is susceptible to CYP-mediated metabolism during absorption. [157]. Englund et al. explored how the expression and activity of transport proteins of the SLC (PEPT1, MCT1, OATPB, OCT1, OCTN2) and ABC family (P-gp, BCRP, MRP2 and MRP3) is expressed in Caco-2 cells in relation to different intestinal regions. Overall, the in vivo–in vitro expression level of transporters was distinctly different, but often highly dependent on a single transporter. For example, expression levels of BCRP were noticeably lower, and MRP2 and OATPB were higher in Caco-2 compared to the duodenum, ileum and colon. For the colon, a substantial difference was also observed for PEPT1. The same group also described how the culturing time can affect the transcript levels; the mRNA level of PEPT1 and OATPB more than doubled between day 4 and day 21, and expression of P-gp was even four-fold higher [169]. It is also noteworthy that there is a large inter- and intra-laboratory-, clone- and passage-dependent variability in metabolizing and transporter proteins [157]. For example, the expression level and functionality of BCRP varies greatly between labs, which might be due to a different origin of the Caco-2 cells [170], and nonstandardized culture conditions [171,172].

*Ex Vivo: Ussing Chambers*

**Figure 3:**
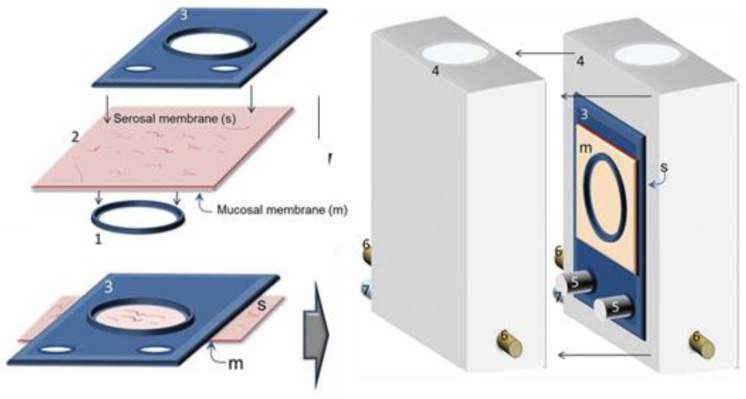
Schematic view of tissue fixation in the Ussing chamber. After the removal of the muscle layer, fat, and sub-mucosa, the tissue clamp is pushed carefully under the intestinal segment. The mucosal side (m) faces the bottom and the serosal membrane (s) faces the top. Then, the tissue holder is pressed down onto the tissue, fixing the tissue in-between the tissue holder and the tissue clamp. After the removal of overlapping tissue, the tissue holder is mounted into the Ussing chamber (4) by fixing it onto the bars (5). The mucosal side can face the left or the right half of the chamber (here the mucosal side faces the left). 6 = connectors for agar-salt bridges; 7 = connectors for carbogen. Adopted with permission from [185], John Wiley & Sons, 2001.

A more complex translational model is the Ussing chamber technique, which comprises tissue, a biopsy (only mucus and mucosa is present), or an epithelial cell monolayer placed between two half chambers, which can be filled with a physiologically relevant solution (Figure 3). The intestinal tissue provides a better representation of the in vivo morphology and physiology (multiple cell types, mucus layer, acid microclimate, surface area), and approximates the in vivo expression levels of enzymes and transporters more closely than several immortalized cell lines [173,174,175,176,177,178], but is donor-dependent. Thus, it is well suited to study both passively and actively transported drugs [179]. The Ussing chamber system has a strong record of predicting the human F_a_ and does so in a robust and reliable manner [175,180,181]. Moreover, the model lends itself to use in various preclinical species, including rats, rabbits, dogs, or monkeys, which can support the interpretation of preclinical in vivo studies [182], but species difference in permeability and metabolic capability can be a restricting factor for human extrapolation [183,184].

When considering colonic uptake of drugs, the Ussing chamber technique allows researchers to evaluate candidate drugs that are intended for extended release, which can prevent attrition in the later stages of drug development. Sjoberg and Tannergren’s group showed a clear sigmoidal relationship between the apparent permeability of human colonic tissue mounted in the Ussing chambers system and the human colonic F_a_, respectively, with both curves being in agreement with one another [61,180]. Sjöberg’s group was also able to report regional differences in drug absorption of passively transported drugs: polar drugs (atenolol, creatinine, mannitol, ximelagatran, rosuvastatin) had the lowest permeability coefficients in the colon (jejunum > ileum ≥ colon), while the reverse was observed for nonpolar and more permeable drugs (antipyrine, oxprenolol, metoprolol and propranolol) for which the highest values were recorded in the colon (jejunum < ileum ≤ colon) [180]. The exact underlying mechanism is unclear, which is why the authors refer to the general physiological differences. It is important to remark that P-gp-substrates can show higher apparent permeability values in the colon compared to other intestinal regions, which is likely due to their lower expression in colonic tissue (see Section 2.1) [186]. Lastly, one should keep in mind that when human tissue is used for permeability assessments, the different medical history, the type of surgery undergone by the donor and the age of the patients can affect the integrity and viability of the resected tissue, and consequently absorption [180].

Lastly, two separate studies [175,184] have shown how including possible drug accumulation in tissue (respectively coined ‘P_*app*_, Total’ and ‘Transport Index’), which is not covered by the conventional apparent permeability, can improve the prediction of the human F_a_. Rozehnal showed that correlation between P*_app_*, _total_ and human F_a_ was only marginally better than P*_app_*, except for metoprolol, ibuprofen, and pravastatin, drugs which significantly accumulated in the mucosa. Miyake showed that the rank order of a 4kDa labelled dextran, atenolol and metoprolol coincided with F_a_ in humans, but only when tissue accumulation was considered. Not factoring in the accumulated drug fraction, which can contribute to absorption at a later stage, would lead to an underestimation of drug permeability. Furthermore, assessing colonic accumulation can be of value since colon-targeted delivery systems aim at local accumulation for an efficacious treatment, while also minimising systemic absorption. Although the low-through-put nature of this technique restricts its application in early stages of drug discovery given the scarcity of human intestinal tissue, it can ameliorate the prediction of drug absorption in the late discovery or early development stage and contribute to the optimization of the lead compounds.

*Organoids*

**Figure 4:**
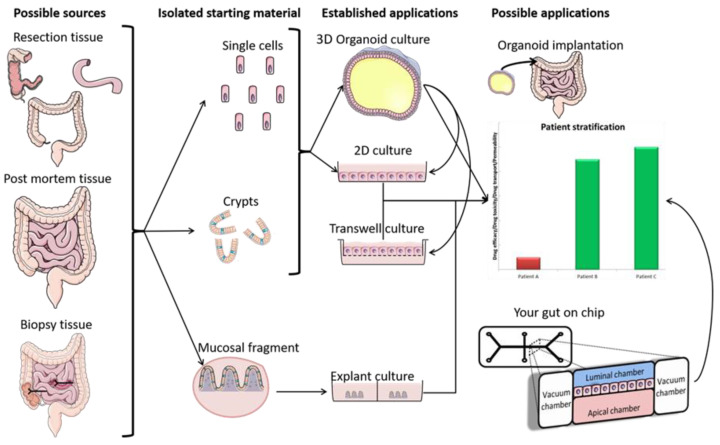
A representation of the generation of 2D and 3D organoids, and their application. Resection-, post mortem-, and biopsy tissue can be used to acquire crypts or single stem cells, which can then be cultured as organoids following described protocols. The 3D Organoids can be exposed to microbes and drugs by microinjection, while the transwell culture (2D) allows both luminal and basolateral exposure. The possible applications are discussed in the text and depicted in the right panel. Adapted with permission from [198], SAGE Publications, 2017.

The establishment of intestinal organoids derived from LGFR5-positive stem cells for long-term culturing and expanding in the presence of growth factors (WNT, R-Spondin and Noggin, all providing the essential stem cell signalling) was described originally for mouse small intestine [187], and later on for mouse colon, human small intestine and human colon [188] (Figure 4). The culture conditions can be modified for enrichment with major epithelial intestinal cells with physiological relevance to biological readouts [189,190]. Those intestinal organoids are grown as 3D cultures embedded in an extracellular matrix (Matrigel).

Xu et al. [191] used hollow 3D organoids with a well differentiated single layer of epithelial cells generated from proximal colon biopsies from patients with CD in remission to study the epithelial integrity of the epithelial organoid culture. Stable gene expression of key junctional proteins such as zonula occludens-1 (ZO-1, also known as tight junction protein 1), occludin and beta catenin were identified in the 3D structure. When applying 2 mM egtazic acid to the epithelial organoid culture, loss in epithelial integrity was observed by migration of a 4-kDa dextran labeled with fluoresceine-isothiocyanate (FITC-dextran 4) into the lumen. This is in agreement with results obtained with Caco-2 cell monolayers using increased FITC-dextran 4 migration and decreased ZO-1 and occluding expression as markers for barrier dysfunction [192]. Those data indicate that tight junctions ensure membrane integrity in organoids and that membrane disruption can be identified using common leakage markers.

Looking at the expression of transporters, Mizutani et al. [193] identified the apical presence of P-gp in 3D small intestinal organoids created from adult mice, and confirmed its functionality by incubating the organoids with a P-gp substrate (e.g., rhodamine123), and an inhibitor (e.g., verapamil). These 3D organoids were reported to be lined by a differentiated epithelial monolayer with the apical membranes facing the luminal space inside. Similarly, Zhang et al. [194] reported functionality of BCRP on the apical membrane of 3D organoids from mouse small intestine, and reported a comparable expression to the small intestinal epithelium of mice. Onozato et al. [195] used human induced pluripotent (iPS) stem cells that were differentiated to functional organoids presenting microvilli structures and tight junctions. Several intestinal cell types like enterocytes, intestinal stem cells, goblet cells, enteroendocrine cells, Paneth cells, and supporting cell types like smooth muscle cells and fibroblasts were also identified. Under these differentiated organoid conditions, the authors demonstrated activity of efflux transporters P-gp and BCRP and the activity of DMEs like CYP3A4. Verapamil and Ko143 inhibited the efflux of rhodamine123 and hoechst33342 transport, substrates of MDR1 and BCRP, respectively. CYP3A4 activity was tested using the substrate midazolam and also observed after induction by rifampicin and 1a,25-dihydroxyvitamin D3. CYP3A4 specificity was further confirmed by inhibition with ketoconazole. Besides efflux transporters, the expression of the uptake transporter PEPT1 was determined by immunofluorescence staining. Other CYP enzymes were not tested for their activity but the mRNA levels of CYP3A4, 2C9, 2C19 and 2D6 were determined and described to be similar to those in the human small intestine. However, it should be noted that all mRNA expression data of functional cell markers, several metabolising enzymes, and transporters were lower than in intestinal tissue. Using adult intestinal stem cells or iPS as the source to generate intestinal organoids can influence the differentiation potential and thus expression and functionality of metabolising enzymes and transporters. It would be interesting how the expression levels generated in those iPS-derived organoids compared to adult stem cell-derived organoids. In addition, the expression and functionality may differ depending on differentiation protocols and might reflect species (e.g., mice and human) and regional differences (e.g., small intestine and colon) as organoids from different species and regions can be generated and compared. This makes the organoid model an attractive model for drug disposition studies not only under “generic” conditions but also to study drug disposition under specific circumstances, as organoids can be generated from any donor e.g., healthy, diseased, geriatric, pediatric, neonatal etc. thereby moving closer to individualized medicine. For example, Dotti et al. [196] have shown the potential to investigate epithelial and mucosal changes of IBD patients in these 3D developed intestinal organoid models. They reported a difference in the regulation of genes associated with antimicrobial defence, secretory and absorptive functions, and gastric phenotypes between cultures expanded from the intestinal crypts of nonIBD controls and patients with UC.

To perform drug disposition studies, drug exposure to the apical and basolateral side is essential. Since traditional 3D organoid culturing techniques only enable direct drug exposure to the basolateral side of the organoid (the apical side faces the inside of the organoid) 2D transwell culturing is essential for permeability studies and has been developed over the past few years. Moon et al. [197] first described transwell culturing conditions for mouse colonic organoids. Subsequently, VanDussen et al. [49] established techniques for transwell culturing of human rectal and ileal organoids as epithelial cell monolayers on membrane inserts. One week after seeding of organoid-derived stem cells on Matrigel-coated transwell inserts, a single layer of intestinal epithelial cells with MUC2-expressing goblet cells and tight junction proteins (ZO-1 and claudin 3) was obtained. The observed mucus layer in this study with a thickness of 36 µm for the rectal monolayer and 26 µm for the ileal monolayer (determined by sedimentation of red fluorescent beads detected with confocal microscopy) may add an advantage to the organoid model as a possible colonic drug disposition model, as discussed earlier (see Section 2.2). As culturing of organoids on transwell inserts as polarized monolayers allows separate access to and sampling from the apical and basolateral compartment, this model may become an interesting tool to evaluate colonic permeability and to explore underlying transport mechanisms similar to the Caco-2 system (see Section 6.2.3.1). As discussed above (Section 6.2.3.1), eEspecially for colon-targeted compounds, the actual tissue accumulation could be of high relevance. By more closely resembling the intestinal physiology than immortalized cell lines, organoids could be a useful novel tool for investigating the tissue accumulation of compounds. However, although transwell culturing of organoids has been described, so far no data related to the expression nor activity of transporters in transwell cultured colonic organoids have been reported.

VanDussen et al. [49] described and Noben et al. [198] reviewed also the development of a monolayer organoid model from different parts of the GI-tract as well as from different disease states (Figure 4). In addition to this and based on their protocol [49], Vancamelbeke et al. [199] used the organoid model to investigate inflammation-induced loss of epithelial barrier function. Therefore, the protocol was optimized to establish monolayers from epithelial cells of non-UC and UC patients.

Although Noben et al. [200] reported that the inflammatory status of the tissues of patients is not maintained when organoids are cultured, the organoid model holds the potential to be able to investigate drug disposition in a diseased (inflamed) stage after induction of barrier dysfunction by adding both tumour necrosis factor alpha and interferon gamma to the culture as reported by Capaldo et al. [201].

The possibility of organoids to be grown on transwell membranes, to create polarized monolayers, the maintenance of integrity, the expression of regional transporters and the presence of mucus producing goblet cells under differentiated conditions, make organoids a promising colonic drug disposition model compared to the traditional models. Additionally, the organoid model provides the possibility to generate, study and compare region-, disease-, and age-specific models which might enable the assessment of drug disposition in specific target populations e.g., IBD patients, pediatric patients, geriatric patients and others. Furthermore, preclinical models can be generated which allow the comparison of preclinical in vivo to in vitro data to further validate the model. However, the characterization of intestinal organoids as a drug disposition model still needs further evaluation.

*Colon Chip*

**Figure 5:**
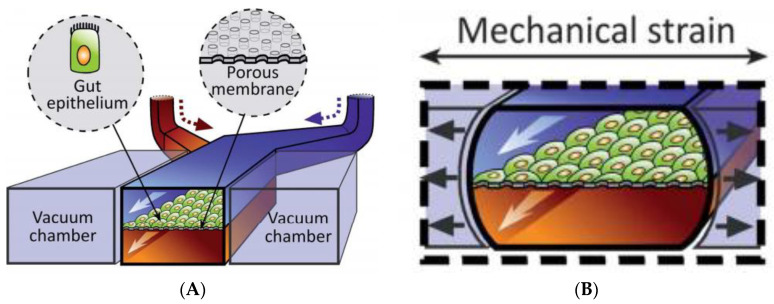
Organ-on-a-chip. (**A**): The central microchannel comprises a flexible porous extracellular matrix (ECM)-coated membrane lined with gut epithelial cells, positioned between two vacuum chambers. (**B**): Mechanical strain is applied to the gut intestinal monolayers by suction from the vacuum chambers. Adopted with permission from [202], Royal Society of Chemistry, 2001.

In the past few years, a number of organ chips, also called microphysiological systems (MPS), have been described for the intestine (Figure 5). The first emerging models especially used Caco-2 as the epithelial cell source [202,203,204]. Organ-on-a-chip separates itself from other systems by incorporating microvasculature, mechanical forces of fluid flow (shear stress) and intestinal peristalsis, though it can be questioned whether this will result in improved absorption and metabolism properties compared to traditional models. Depending on the provider, those chips typically consist of two layers of microfluidic channels separated by either a thin porous membrane (polydimethylsiloxane (PDMS)) coated with an extracellular matrix (ECM) on top of which Caco-2 cell are plated [202,203,205] or by ECM only on which Caco-2 cells are seeded in one lateral line forming a polarized epithelial tube [204]. The culture medium is then perfused through both channels and, thus, the apical and basal sides are accessible for drug exposure.

The use of shear stress (flow) demonstrated some advantages over traditionally used transwell culturing, especially regarding differentiation and polarization, which is achieved in Caco-2 chips as early as 3–4 days after cell seeding in comparison to the 21 days of culturing needed for traditional transwell monolayers [204,205,206]. Applying shear stress not only decreased the differentiation time tremendously but also increased the cell size and polarization status (epithelial cells were 6-fold taller in size vs. transwell culturing with clear basal nuclei) compared to static transwell Caco-2 cells presenting rather flattened, almost squamous epithelial cells [202,203]. The addition of mechanical stress (cyclic mechanical strain mimicking peristalsis) in particular enabled the spontaneous formation of undulations and folds exhibiting the morphology of intestinal villi, and intestinal differentiation. However, similar to the traditional static monolayer, the cells are somewhat more representative of the small intestine concerning the observed cell types (enterocytes, goblet cells, enteroendocrine cells, and Paneth cells) [202,203]. However, in contrast to static cultures, the goblet cells present in the Caco-2 chip have been shown to produce MUC2. Interestingly, peak transepithelial electrical resistance (TEER) levels were 3–4 fold higher in the Caco-2 chip under flow conditions than in static transwell cultures, no matter whether mechanical stress was applied or not. However, the *P_app_* value for fluorescent dextran only increased (4-fold) in the presence of mechanical stress [202], implying that cyclic mechanical strain might affect paracellular transport mechanisms. Despite those interesting observations, it was not clearly shown that main drawbacks of the Caco-2 system like the lack of CYP3A4 expression (increased activity was shown by Kim et al. [205] but only for a very limited data set), deviating expression of uptake and efflux transporters from human intestinal tissue, large clone- and passage-dependent variance, and increased monolayer integrity compared to the in vivo situation, could be overcome by incorporating Caco-2 cells in a more physiological environment [202,203,204,205].

Recently, a colon-chip using colonic organoids from the sigmoid and ascending region as the epithelial cell source has also been described [50], with specific focus on mucus production. The authors demonstrated that maturation and differentiation to goblet cells in the colon-chip occurred spontaneously under stem cell expansion conditions. This is not observed in “traditionally cultured” organoids in Matrigel or on transwell inserts, which require differentiation medium, depleting the stem cells [49,188,207]. Similarly to the Caco-2 chip, when using colonic organoids, a polarized columnar epithelium with undulating structures was formed (only shear stress, no mechanical stress was applied), and the appearance of ZO-1 containing tight junctions and F-actin rich brush boarders was confirmed. Most striking were the high levels of MUC2 producing goblet cells, similar in morphology and number (approximately 15% of all cell types) to those seen in histological human colon sections (approximately 10–30% dependent on donor) which were observed in the colon organoid chip. Moreover, those functional and differentiated goblet cells produced a mucus layer that was comparable to in vivo colonic bilayered mucus concerning thickness and structural organization into a penetrable (for bacteria) outer and impenetrable (for bacteria) inner layer.

In summary, a number of organ-on-a-chip models are available. Several chips use Caco-2 cells as the epithelial cell source. Although some advantages over traditional transwell culturing could be identified, especially concerning maturation, differentiation, and mucus production, most Caco-2 specific drawbacks remain even when using chip-technologies. However, the recently described colonic organoid chip model appears to be a very promising tool for colonic drug permeation studies due to its physiological representation of bilayered mucus. Additionally, the chip system allows flexibility concerning the intestinal epithelial source. Thus, different patient and age populations, as well as preclinical species and different intestinal regions could in principle be addressed. Although the intestine-chip is a highly physiological system, it comes with some drawbacks, especially concerning high-throughput usage, cost-efficiency, and assay simplicity (flow rate needs to be taken into consideration). A further functional characterization would be of high interest.

##### 6.2.3.2. Available Small Intestinal Models

Scaffold-Based Systems

*RepliGut* ^®^

**Figure 6:**
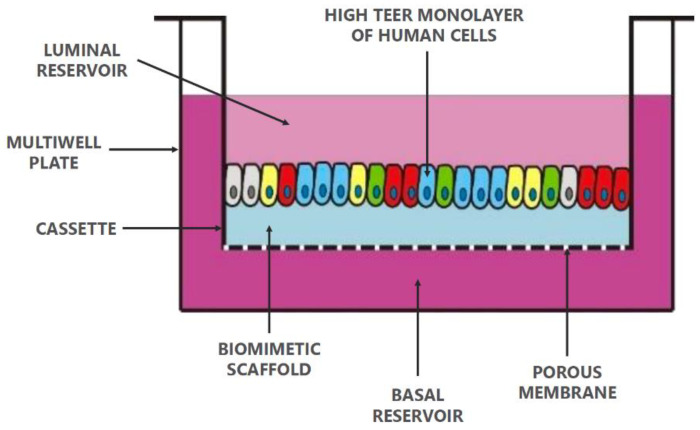
RepliGut**^®^** planar growing primary epithelial cells on a biomimetic scaffold. Adopted with permission from [210], Altis Biosystems.

A promising model is RepliGut**^®^**. Appyling mammalian stem cell technology that uses a collagen hydrogen scaffold a polarized and self-renewing planar monolayer culture of colonic epithelial cells is recreated (Figure 6). These primary tissue derived monolayers contain all colonic cell types (including goblet, enterocyte and enteroendocrine cells), express adherens and tight junctions (localized ZO-1, E-cadherin, and occludin), and have microvilli [208]. The option also exists to use a 3D model that accurately captures the colonic crypt structure, which is polarized with a gradient of growth factors. An advantage of the monolayer format is that both the luminal and basolateral surfaces are accessible, allowing drug permeability studies. The model is furthermore available from various regions and different donors, which allows region-dependent drug absorption studies, disease modelling and microbiome research [209]. Although promising, the characterization of absorption, distribution, metabolism, and excretion (ADME) is not as advanced.

*EpiIntestinal™ (MatTek)*

**Figure 7:**
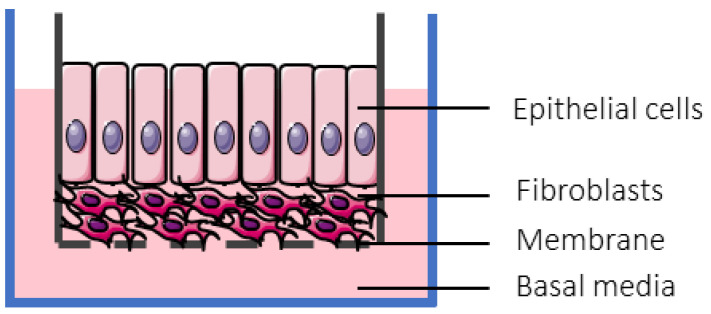
EpiIntestinal full thickness tissue cultured at the air-liquid interface. Adopted with permission from [159], Oxford University Press, 2018.

EpiIntestinal^TM^ (MatTek, Slovak Republic) is a novel organotypic 3D microtissue, which is currently available as a small intestinal model, derived from the ileum of a 19-year old female donor. Having this microtissue platform developed for other intestinal regions holds a great promise for colonic drug delivery research in the future. EpiIntestinal^TM^ can be used in two different setups, either as an epithelial layer only (EpiIntestinal^TM^) or in co-coculture with human fibroblasts, which are commercially purchased and thus derived from a different donor (EpiIntestinal-FT™). The epithelial cell layer is cultured at the air-liquid interface (ALI) on a transwell insert, thus creating a bicompartmental setup with an apical and a basolateral side (Figure 7). Several reports have been published recently describing EpiIntestinal™ as a model to assess intestinal drug disposition [2,157] and safety [159]. Ayehunie et al. [2] performed a comprehensive characterization of EpiIntestinal™, zooming in on structural features, drug transporters and metabolizing enzyme expression, and functional evaluation of the bidirectional transport of 11 test drugs. The TEER values measured with EpiIntestinal™ (152.5 ± 39 Ω*cm^2^) are in close proximation to human intestinal ex vivo tissue (50–100 Ω*cm^2^) indicating that structural features may more closely mimic small intestinal tissue than Caco-2 cell monolayers with TEER values ranging from 250–2400 Ω*cm^2^ [211]. This is further underpinned by microscopic imaging demonstrating a crypt–villi architecture, positive cytokeratin-19 (epithelial differentiation marker), villin (enterocytes marker), and vimentin (fibroblast marker) staining as well as transmission electron micrographs confirming brush boarder membranes and tight junctions in EpiIntesintal™ tissue. Given the crypt–villus architecture and the presence of microvilli, the surface area might be enlarged compared to traditional 2D cell culture models. Functional characterization was performed, using 11 reference drugs (BCS I–III, with the majority of drugs being BCS III), by determining P_*app*_ and comparing those values to Caco-2-derived data as well as to the known human oral bioavailability of those drugs. For EpiIntestinal™, P_*app*_ values correlated more closely with known human absorption (R^2^ = 0.91) than for those obtained with the Caco-2 model (R^2^ = 0.71), most likely due to the closer in vivo-like structure, barrier function, and transporter expression as suggested by the authors. EpiIntestinal™ appears also suitable to study transporter-dependent permeation. qPCR data confirmed the expression of *ABCG2*, *ABCC1*, *ABCC2*, and *ABCB1* encoding BCRP, MRP1, MRP2, and P-gp, respectively. Unfortunately, a direct comparison to the explant tissue as given for expression data of metabolising enzymes is lacking. However, functional transporter activity was confirmed using P-gp, BCRP, and MRP2 substrates and transporter specific inhibitors. Efflux ratios > 2 were reached for all transporter substrates and efflux decreased substantially in the presence of a transporter-specific inhibitor. The expression level and functional activity of several important uptake transporters (including OCT1, OATPs, and PEPT1) was not evaluated, and requires further investigation. Finally, the expression and functional activity of intestinal CYP450 DMEs (CYP3A4, CYP3A5, CYP2C9, CYP2C19) were also confirmed by Ayehunie et al. [2] and were further investigated by Cui et al. [157]. The study described by Cui and coworkers mainly focused on characterizing EpiIntestinal^TM^ as an in vitro tool to predict the human GI first pass availability of drugs (F_a_ × fraction escaping enterocyte metabolism (F_g_)) because both absorption and metabolism can be determined using EpiIntesintal^TM^. They convincingly show that GI first pass availability determined with EpiIntestinal^TM^ is in good correlation with F_a_ × F_g_ in humans for the 12 reference drugs (a range of low, medium and high bioavailability, mainly metabolized by CYP3A4) used in the study. Moreover, they provide metabolic activity data for CYP3A4, CYP2B6, CYP2C8, CYP2C9, CYP2C19, CYP2D6, CYP2J2, UGTs, SULTs, and Carboxylesterase 1 and 2 (CES 1 and 2) in comparison to Caco-2. Interestingly, despite confirming the well-described lack of CYP3A4 activity in Caco-2, other CYP enzymes like CYP2C9, CYP2C19, and CYP2D6 could be detected in Caco-2 and differed only slightly from EpiIntestinal^TM^. Although the activity of SULT and UGT1A1 was 3–4-fold higher in EpiIntestinal^TM,^ it could also be detected in Caco-2. Surprisingly, and differently to tissue abundance [212] with high CES2 and low CES1 presence in the intestine, CES1 and CES2 demonstrated similar activity in EpiIntestinal™ [157], using Dabigatran etexilate as a substrate. However, Dabigatran etexilate is also subjected to substantial P-gp mediated efflux, which might limit drug availability for both enzymes. Therefore, to more objectively conclude on CES1 and CES2 activity, the use of additional CES1 and CES2 substrates for validation might be helpful. Given the results of the above-described studies, it seems evident that EpiIntestinal^TM^ could also be applied to evaluate the intestinal safety of drugs since e.g., metabolite-mediated toxicities, or transporter-mediated drug–drug interactions could potentially be identified with EpiIntestinal^TM^. This has been elegantly demonstrated by Peters and coworkers [159] employing a validation set of 39 drugs with known diarrhea responses in humans. The authors used the TEER measurement as a functional readout for diarrhea. When TEER values were adjusted for clinical exposure, a threshold could be established distinguishing drugs that induce diarrhea from those which do not.

In summary, this ileal 3D microtissue is attractive for studying intestinal permeability, metabolism, DDI, and the safety of drugs due to its structural and physiological analogy with the small intestine, its polarized orientation with access to apical and basolateral compartment, its potential to closely predict F_a_ × F_g_, its long-term culturing capacity (up to 6 weeks), and the availability of different setups (6-well, 24-well, 96-well). The model would benefit from the characterization of uptake transporters, and clarification on potential differences between the full-thickness and the epithelial layer-only tissue. Currently, this system is available from one specific donor, which, especially for metabolic studies, will not be representative of the total target population. Having this engineered 3D tissue available from other intestinal regions, e.g., the colon, as well as from preclinical species would be advantageous.

*3D Bioprinted Small Intestinal Tissue (Organovo)*

**Figure 8:**
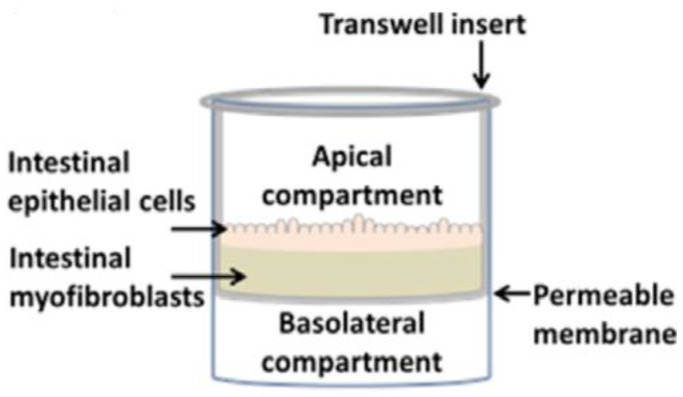
The 3D small intestinal microtissue engineered utilizing bioprinting of an interstitial layer containing adult human intestinal myofibroblasts followed by adult human intestinal epithelial cells creating a bilayered architecture. Adopted with permission from [3], Elsevier, 2018.

Generated using bioprinting (Organovo 3D NovoGen Bioprinter system), this 3D small intestinal (ileal) microtissue (Figure 8) also appears suitable for drug absorption and stability profiling [3]. Similar to previously discussed bioengineered microtissues, it exhibits polarized columnar epithelial morphology supported by a fibroblast layer with a brush boarder and tight junction formation, and the presence of specialized cells like enterocytes, goblet cells, Paneth cells, and enteroendocrine cells. Gene expression data demonstrated the presence of the major intestinal Phase I (CYP3A4, CYP2C9, CYP2C19, CYP2D6, CYP2J2) and some Phase II (only UGT1A1 and GSTP1) metabolising enzymes as well as major efflux (P-gp; BCRP) and uptake transporters (PEPT1; OATP2B1) which more closely represented native intestinal tissue expression as compared to Caco-2 monolayers. Further studies using luminogenic P450 substrate conversion confirmed the activity of CYP3A4 and CYP2C9, as well as CYP3A inducibility with rifampicin and inhibition with ketoconazole. Likewise, the 3D tissue correctly distinguished a small set of low-, intermediate- and high permeability drugs, supported by a strikingly physiological TEER value of 50–100 Ohms*cm^2^ [211] which could be maintained during a 10–21 day culturing period. Finally, P-gp and BCRP-activity was confirmed using digoxin and topotecan, respectively, as substrates which yielded efflux ratio >2. Those efflux ratios were reduced <2 in the presence of specific P-gp (Zosuquidar) and BCRP (Ko143) inhibitors.

The 3D small intestinal tissue described by Madden et al. holds great potential for permeability (apical and basolateral side accessible), metabolism and safety (not discussed in this review) profiling of drugs, although the model would benefit from establishing a correlation for first-pass GI availability in relation to humans. Other promising features are that different donors and, potentially, other intestinal regions (already described by Madden et al. [3]) can be used. However, user-friendliness, platform-format, and availability can be of limiting nature and need further clarification.

*Primary Enterocyte Systems*

The primary enterocyte systems marketed by the company in Vitro ADMET Laboratories (iVAL, Colombia, MD, USA) can be categorized into (1) enterocytes, (2) MetMax™, and (3) Cryopreserved Human Intestinal Mucosa (CHIM™). All systems are derived from the small intestine; a colon model is currently not available. The first described system [213], cryopreserved enterocytes, are human (other species are available) derived primary enterocytes which have been isolated, digested with collagenase, partially purified by differential centrifugation, and finally cryopreserved. MetMax^TM^ enterocytes are permeabilized enterocytes with maximized metabolism (proprietary methodology, preserved metabolic pathways of the endoplasmic reticulum, cytosol, mitochondria, lysosomes, and plasma membrane according to manufacturer), which require the addition of cofactors to determine metabolic enzyme activity [214]. The latest addition to those primary enterocyte models is CHIM™. CHIM™ has been generated by applying collagenase digestion to intestinal tissue but digesting only to intestinal villi and not single cells, followed by partial purification using differential centrifugation, gentle homogenization, and finally cryopreservation in a proprietary medium [215]. All three systems are easy to use (suspension incubation), available from humans as well as preclinical species, and can be derived from specific donors or donor pools. Moreover, the CHIM^TM^ technique is available from duodenum, jejunum, and ileum, allowing regional comparison of DME activity. However, none of the systems are suitable for long-term culturing (enterocytes sustain maximally for 4 h, CHIM^TM^ for 24 h), for permeability studies (no polarized epithelium), or for safety assessment (limited capabilities of CHIM^TM^). In cryopreserved enterocytes, CYP2C9, CYP2C19, CYP3A4, CYP2J2, CES2, UGT, and SULT activities could be confirmed [213] but were subject to substantial interindividual differences and in general low compared to MetMax™ and CHIM^™^. This might be due to substantial viability improvement for CHIM^TM^ and cofactor supplementation for the MetMax™ system in comparison to the enterocytes only. While MetMax™ demonstrates good enzyme activity for the main intestinal CYP450 enzymes, UGT, and SULT, which is in concordance with gene and protein expression data concerning the ratios/representation of the different enzymes [27], CHIM™ is an intact enterocyte system (no cofactor supplementation) yielding strikingly high metabolic activity of CYP450 and Phase II enzymes [215]. Interestingly, CYP2D6 activity was not detected in all three enterocyte systems, whereas activity in EpiIntestinal™ (see Section 6.2.3.2) was comparable to CYP2C19 (2% of CYP450-pie chart), which is similar to proteomics abundance data from intestinal jejunal and ileal tissue [28]. This might, however, be a result of donor- or region-dependent differences. CHIM™ is also suitable for CYP-induction studies and can be used to identify region-dependent metabolism from the same and different donors, with the jejunum showing the highest metabolic activity and the ileum generally showing the lowest activity of the small intestinal regions in the study performed by Li et al. [214]. In general, normalization to protein concentration would enable a better comparison of enzyme activities among the different systems. This was considered in a very recent study by Davies and coworkers [216]. In this comprehensive study, the authors compared intestinal microsomes, MetMax™, and CHIM™ in their capability to accurately determine F_g_ of a diverse set of 32 drugs, including substrates for oxidoreductive, hydrolytic, and conjugative metabolic pathways. They demonstrated that all three models show reliable CYP450 and UGT activities but MetMax™ and CHIM™ might offer advantages in estimating low clearance drugs and drugs metabolized by e.g., sulfation, hydrolases, flavin-containing monooxigenases, and other alternative pathways [216]. Li and coauthors [215] also suggest CHIM^TM^ as a model to evaluate the enterotoxic potential of drugs. However, concentration-dependent toxicity was only shown for two drugs demonstrating relatively high IC_50_ values and lacking comparison to in vivo exposure. Moreover, the use for safety assessment is substantially limited by the maximum culturing time of 24 h.

Altogether, the enterocyte systems are well-suited for the determination of enteric metabolism, they are easy to use, can be selected according to intestinal regions of interest and donor demographics or used as pooled samples to represent a large variety of the patient population. They are available from preclinical species and thus enable in vitro/in vivo comparison. Moreover, CHIM^TM^ system enables DDI studies as CYP enzymes can be induced. MetMax™ might be especially beneficial to assess metabolism for poorly permeable drugs and drugs with slight cytotoxicity [216] due to its increased permeability. Due to the restricted sustainability and suspension-based nature of these models, toxicity testing, and permeability assessment have clear limitations.

*Small Intestinal Chip*

**Figure 9:**
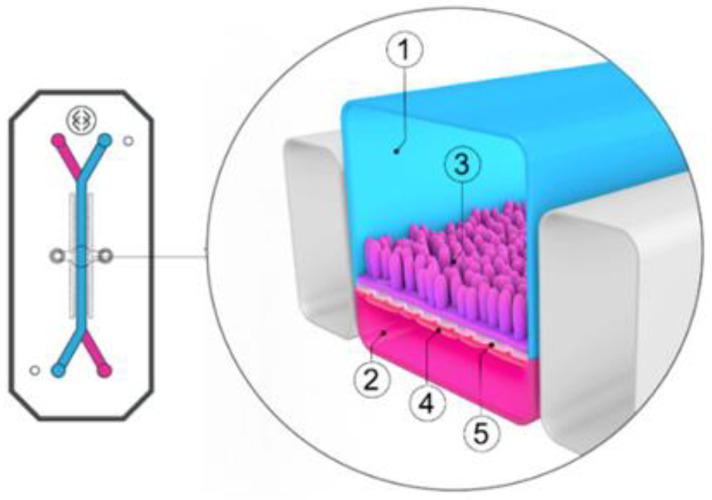
Organ-on-a-chip. Duodenum Intestine-Chip from Emulate, including its top view (**left**) and vertical section (**right**) showing: the epithelial (1; blue) and vascular (2; pink) cell culture microchannels populated by intestinal epithelial cells (3) and endothelial cells (4), respectively, and separated by a flexible, porous, ECM-coated polydimethylsiloxane (PDMS) membrane (5). Adopted from [158], eLife, 2020.

Here, we focus on reviewing the duodenal chip commercialized by Emulate (Boston, USA) for which basic characterization as an ADME tool has been performed [158]. The duodenal chip consists of epithelial cells derived from adult intestinal stem cells (crypts) that were acquired by the enzymatic dissociation of organoids and then seeded into the microtube, and primary intestinal microvasculature endothelial cells (HIMECs, different donor) seeded into the “vascular” channel of the duodenal chip (Figure 9). Medium flow was applied to both channels and once confluency was reached, cyclic mechanical strain was applied in order to mimic intestinal peristalsis. This setup resulted in accelerated polarization (in 72 h), the formation of villuslike structures, and apical microvilli. Shear stress in comparison to static cell culture conditions boosted cell maturation and differentiation into enterocytes, goblet cells, enteroendocrine cells and Paneth cells with cell ratios closely reflecting in vivo ratios. The authors further characterized the chip by applying differential gene expression analysis to intestine-chips generated from intestinal organoids and comparing those data to the intestinal tissue and organoid source. Both duodenal organoids and intestine-chips used for differential gene expression analysis were first expanded for 6 days followed by 2 days of differentiation. Interestingly, although the duodenal organoids and the duodenal chip derived from the same tissue source, 472 genes were identified with significantly different expression levels. Moreover, 305 genes, associated with digestion, transport of nutrients and ions, extracellular matrix organization, wound healing, metabolism, and tissue development, were expressed similarly in the duodenal chip and the tissue of origin but were differently expressed in the organoid cultures. While those data strongly suggest that the duodenal chip transcriptome more closely resembles the human adult duodenum, it should be taken into consideration that the differentiation and maturation in the chip is enhanced and, therefore, when comparing to 2-day differentiated organoids, the organoid culture might not yet be fully matured. Further evaluation of the duodenal chip confirmed apical expression of MDR1, BCRP1, and PEPT1 gene expression comparable to the tissue of origin and functional activity of MDR1, demonstrated by increasing Rhodamine 123 cell accumulation in the presence of the MDR1 inhibitor vinblastine. Finally, CYP3A4 metabolism using testosterone as a substrate, and CYP3A4 induction by rifampicin and 1,25-dihydroxyvitamin D3 could also be demonstrated.

The duodenal chip (organoids from adult donor) described by Kasendra et al. is a follow-up of their previously described duodenal chip using pediatric donors [158]. The authors convincingly show that the addition of a microvascular channel, shear stress, and mechanical strain mimicking peristalsis creates an intestinal microenvironment which appears to closely resemble the in vivo situation, especially as demonstrated with gene expression data, imaging techniques, and some functional data.

#### 6.2.4. Colonic Microbiome-Based In Vitro Tools

The use of single enzymes or a combination of enzymes, as well as the inclusion of fecal or cecal material in incubation media to explore colonic drug and formulation behavior have been mentioned in Section 6.2.1.4. In several multicompartmental in vitro tools, which mimic the GI tract or GI tract fluids, the microbiome is included, which allows for studying the interaction between the drug and the human gut microbiome in isolation. Gut microbiome models include the Netherlands Organization for Applied Scientific Research (TNO) dynamic model of the human large intestine (TIM-2) [5], and the Simulator of the Human Intestinal Microbial Ecosystem (SHIME^®^) [4]. However, these models do not permit communication between bacteria and intestinal epithelium. Based on microbiome studies, new comprehensive approaches in which the interplay between the microbiome or a microbiome component and the epithelial lining is included are emerging. Therefore, the microfluidics based model HuMix (human–microbial crosstalk) [6] and the microbiome intestine-chip [6] will also be discussed in this section.

##### 6.2.4.1. TNO Intestinal Model (TIM)

The TNO Intestinal Model (TIM) can be used to simulate intraluminal drug behavior in a dynamic setting, as various parameters (mixing, meal transit, secretions, variable pH, contents of GI fluids, the variable amounts of digestive compounds, and water) can be remotely adjusted and controlled to represent different species, age, pathologies and meals. So far, one colonic model (TIM-2) and three different upper GI-tract TIMs have been developed: TIM-1, TinyTIM and TIM-agc. TIM-1 consists of four compartments (stomach, duodenum, jejunum and ileum) that are connected by peristaltic valve pumps, whereas TinyTIM only has a single small intestinal compartment and no ileal efflux. The advanced gastric compartment (TIM-agc) can simulate gastric tone and reduction in gastric volume in comparison to TIM-1 where homogenized gastric contents are obtained. While TIM-1 is the most frequently used setup, the TinyTim setup benefits from a higher throughput because of its simplicity [217].

**Figure 10:**
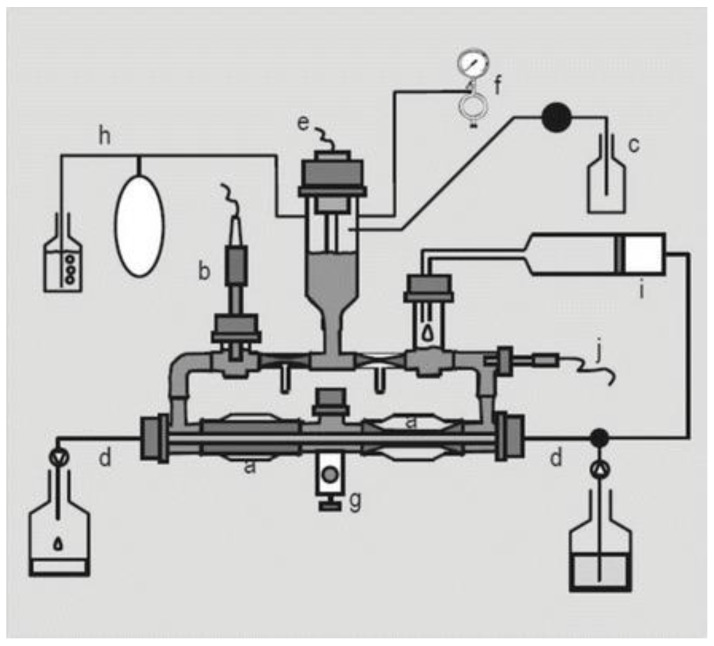
Schematic overview of the Netherlands Organization for Applied Scientific Research (TNO) in vitro model of the colon (TIM-2). (a) peristaltic compartments containing fecal matter; (b) pH electrode; (c) alkali pump; (d) dialysis liquid circuit with hollow fibre membrane; (e) level sensor; (f) N_2_ gas inlet; (g) sampling port; (h) gas outlet; (i) ‘ileal efflux’ container; (j) temperature sensor. Adopted from [5], SpringerLink, 2015.

The TIM-2 model (Figure 10) simulates colonic conditions and makes use of fecal incubations. As such, the model can assess interindividual profiles (various fecal donors, or pooled), demographics (age, pediatric and geriatric population) and healthy or diseased state (IBD) [5]. However, so far TIM-2 has especially been used to explore food effects. Although the TIM-2 model has the potential to evaluate colonic drug disposition taking into account an altered microbiome due to diet and diseased state, no in vitro studies exploring the effect of the microbiome on the solubility, dissolution, stability and bacterial degradation of drugs have been performed.

The TIM-2 model contains a level sensor to control the volume and makes use of nitrogen to create anaerobic conditions. Samples can be collected through the sampling-port or from the dialysate. Formed gas can escape through the bubbling-vial. The ‘ileal efflux’ container is a syringe through which the microbiome is fed into the system; it contains a simulated ileal efflux medium, which mimics the luminal composition of the ileal-cecal region [218,219,220].

TIM-2 uses peristaltic movements which allow better mixing compared to stirring or shaking. The system is kept at a pH of 5.8, mimicking the pH in the proximal colon (pH 5.8–6.8 fasted status [62]); 1M NaOH is secreted to neutralize the acids produced by the microbiome. A dialysate system is included in the system to remove accumulating microbial metabolites, which otherwise would lead to death of microbes and thus changes in the microbiome. This system can maintain an active microbiome for 3 weeks.

Time-dependent sampling is achieved by sampling the lumen through the sampling-port or by collecting the dialysate. This frequent sampling allows studies to investigate the kinetics of the production of microbial metabolites and allows researchers to make an almost complete mass balance. As mentioned, the pH is usually standardized at pH 5.8 to mimic the proximal colon. However, it is also possible to mimic dynamic pH changes in the entire colon as the pH can be programmed as a gradient over time: a pH of 5.8, 6.4 and 7.0 can be used representative for the proximal colon, transverse colon and distal colon, respectively (see Section 3.2). This setup is crucial for a full assessment of colonic drug behavior of relevant colon targeting formulations.

Using TIM-2, it was shown that the microbiome quickly responds to changes in diet [221]. A high carbohydrate diet was compared to a high protein diet and resulted in modification of the activity and composition of microbiome. This implies that a standardized diet should be followed by the subjects before collecting human fecal content. The alteration of the microbiome in diseased states has been discussed in Section 4. Although TIM-2 holds the potential to assess diseased state conditions (e.g., fecal contents from IBD patients), this has not yet been tested nor compared with heathy microbiome.

The main disadvantages of the TIM system are its complexity and low throughput. Its advantages are the possibility to test multiple parameters in parallel (e.g., pH and microbiome activity), to monitor one single parameter, and the presence of a dialysis system and peristalsis. While TIM-2 has so far been used to investigate food effects, it has the potential to evaluate colonic drug solubility, dissolution, and bacterial degradation using either large microbiome pools or individualized microbiome (diet, healthy, diseased state, different age groups, etc.).

##### 6.2.4.2. SHIME^®^

**Figure 11:**
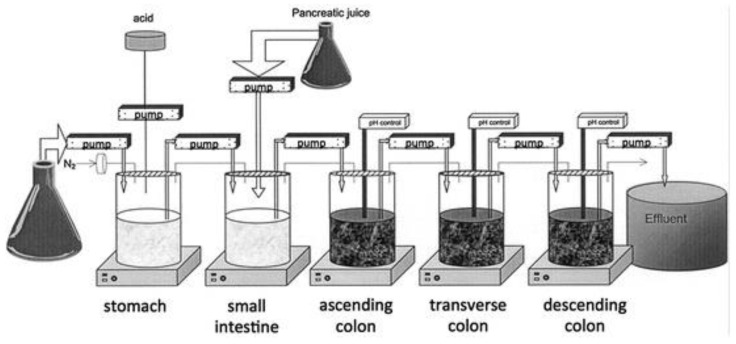
Schematic overview of SHIME^®^. Adopted from [4], SpringerLink, 2015.

SHIME^®^ (Simulator of the Human Intestinal Microbial Ecosystem) was developed in 1993 [222] and consists of five compartments (stomach, small intestine, ascending colon, transverse colon and descending colon) (see Figure 11) that are interconnected with peristaltic pumps. This multicompartment dynamic simulator was created once it was established that the fecal microbiome used in single-stage models differs from the in vivo colon microbiome in terms of pH, redox potential, available nutrients and microbial contents. As these environmental parameters change dynamically, it is impossible to simulate a representative bacterial culture in one compartment.

The setup is extensively discussed by Van de Wiele et al. [4]. Briefly, all double-jacketed glass vessels operate at 37 °C and are kept anaerobic using N_2_ gas or a 90/10% N_2_/CO_2_ gas mixture during the total length of an experiment (up to several days). Subsequently, defined nutritional medium and bile liquid are added by a fill-and-draw principle to the first (stomach) and second (small intestine) compartment, respectively. This medium is composed of a mix of carbohydrate, protein sources, mucins, minerals and vitamins [222]. Upon digestion in the first two compartments, the slurry is pumped into the third (ascending colon) compartment where colon digestion is initiated. This slurry is constantly mixed with magnetic stir bars. The three colon compartments (ascending colon, transverse colon and descending colon) are monitored for constant volume and pH control. Retention times for the upper GI-tract and lower GI-tract compartments are modulated by flow rates and compartment volumes, respectively. The SHIME^®^ set-up is completely computer controlled, therefore different pH profiles can be established. The pH of the gastric and small intestinal compartments is typically fixed at a pH of 2.0 and a slightly acidic to neutral pH, respectively. The following three colon compartments are fixed at a pH of 5.6–5.9, 6.1–6.4 and 6.6–6.9, respectively.

The relevance and validation of the model have also been tested. Metabolism of the prodrug sulfasalazine by intestinal bacteria to the active metabolites sulfapyridine and mesalazine (5-aminosalicylic acid) was tested and was consistent with in vivo data gathered by Peppercorn and Goldman [223]: no conversion into the active drug 5-aminosalicylic acid was detected in the gastric compartment, limited conversion in the small intestine compartment and full conversion in the colon compartments.

The SHIME^®^ model consists of different features [4]. Briefly, the model benefits from the regulated addition of nutritional medium to the stomach, and pancreatic and bile liquid to the small intestine. Moreover, the transfer to and the retention times within the colon compartment can be varied by the flow rate and the compartment volume itself. An important feature of the SHIME^®^ model is its flexibility; it is simple to add or remove compartments depending on the kind of study to be performed. Another important feature is the possibility to change the target group: adult vs. infant, healthy vs. diseased, as well as preclinical species (e.g., pigs, dogs). In that case the following will be adapted: microbial inoculum, residence time, contents of gastric juices, pH, feed, feeding regimes and temperature. A last feature was revealed by a study of Possemiers et al. [224]. They reported that the microbiome from an 8-prenylnaringenin producing volunteer and nonproducing volunteer could be stably transferred to the SHIME^®^ model and that the metabolic phenotype was preserved.

**Figure 12:**
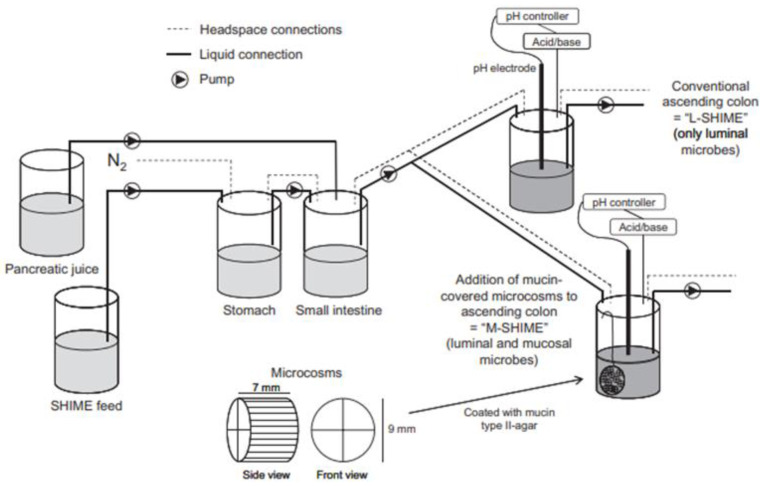
Schematic overview of the implementation of a new second unit modified by incorporating a mucosal compartment (=mucosal SHIME or M-SHIME), which contained 100 mucin-covered microcosms per 500 mL suspension. The general experimental design is composed of several double-jacketed vessels, simulating the stomach, small intestine and three main colonic regions. In this experiment, only the first colon compartment (ascending colon) was used and inoculated with a human fecal microbiome. The first ascending colon unit consisted of the conventional set-up that only harbors luminal microbes (=luminal SHIME or L-SHIME), whereas the second unit is the M-SHIME. Adopted with permission from [226], John Wiley & Sons, 2011.

The inoculum used in the SHIME^®^ model consists of fecal microbiome as the different parts of the human colon region are difficult to access. Depending on the research question, it can be preferred to use individual fecal material instead of pooled material so that individual microbial metabolic phenotypes are maintained. This is of interest when research groups want to investigate interindividual microbial differences. Nevertheless, pooled fecal material can be used when the general effects of fecal material are being determined (e.g., to assess the stability of a drug). Besides interindividual fecal variability, the mucosal microbiome is known to differ in composition from the luminal microbiome (see Section 2.3) and to be responsible for the colonization of the mucus overlying the gut epithelium. Due to its proximity to the epithelial cells, the mucosal microbiome is thought to play an important role in the modulation of gut health [225]. Given the fact that mucosal microbiomes differ in composition and that the mucosal environment is poorly accessible, Van den Abbeele et al. [226] developed mucosal SHIME (M-SHIME^®^) to obtain a better understanding of the importance of a possible host–microbe interactome in a gut simulator model. This first feature was achieved optimizing SHIME^®^ by enabling colonization of the mucosal microbiome using microcosms submerged in mucin agar and combined in a polyethylene ring, added to each colon unit and followed by inoculation with fresh stools (Figure 12) [226].

Van den Abbeele et al. [227] showed a higher abundance of butyrate producing Clostridium clusters IV and XIVa in the microspheres of M-SHIME^®^, responsible for the delivery of butyrate as primary energy source to the colonocytes and strengthening of the tight junctions, crucial to maintaining gut barrier integrity in vivo. In addition to the use of individual inocula, Van den Abbeele et al. [227] determined that M-SHIME^®^ was able to maintain the mucosal composition of an individual and revealed that a high variability in the microbial dataset was obtained due to in vivo/in vitro transition and the difference between the luminal and mucosal contents. Van den Abbeele et al. also proved that the addition of a mucosal environment allowed the colonization of specific microbes (e.g., *Lactobacillus mucosae* and *Lactobacillus rhamnosus GG*) which is in correspondence with the in vivo situation. These data reveal the need for an in vitro assessment of the mucosal microbiome.

The M-SHIME^®^ model can be used to investigate the effect of therapeutic treatments or diseased state on the functionality or composition of the microbiome. Vermeiren et al. [228] reported a depletion in butyrate producing microbial communities in both luminal and mucosal samples from UC patients compared to healthy volunteers. Vanlancker et al. [229] revealed that two chemotherapeutics (5-fluorouracil and irinotecan) did not cause significant changes in the functionality and composition of the colon microbiome. El Hage et al. [230] demonstrated that a decrease in propionate concentrations, upon antibiotic-induced microbial dysbiosis, could be restored after incubation with a propionogenic consortium. Looking in the opposite direction, so far no data are available in the public domain concerning the effect of the in vitro microbiome on the stability of colon-targeted drug delivery systems, although the set-up seems to have the interesting potential to do so.

##### 6.2.4.3. HuMix

**Figure 13:**
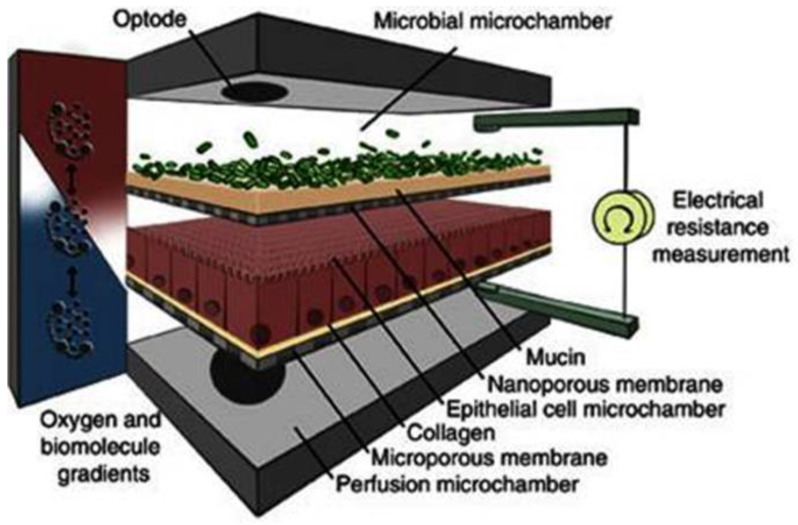
Schematic overview of the human–microbial crosstalk (HuMiX) model including human epithelial cells and gastrointestinal microbes. Adopted from [6], Springer Nature, 2016.

Recently developed intestinal in vitro models specifically aim to be more representative of the in vivo gut conditions. One focus area of emerging in vitro tools is simulating the gut host–microbiome interaction, as dysbiosis can be associated with intestinal diseases; e.g., CD and UC. Mimicking the interplay between human gut microbiome, epithelial and immune cells is, however, challenging. Epithelial cells need to be viable, differentiated, and have a relevant permeability, whilst the microbial feature needs to capture a physiological composition, substrate utilization and metabolite production (such as SCFAs). The main obstacle, however, lies in the different oxygen-requirements; gut bacteria are obligate or facultative anaerobes in contrast to the aerobic epithelial cells. Recently, Wilmes’ group has succeeded in introducing this feature in HuMix (human–microbial crosstalk), an aerobic-anaerobic coculture system and modular microfluidic device (Figure 13) [6]. Their studies validated that these distinct oxygen levels were indeed captured between the microbial microchamber (although not completely anaerobic) and the epithelial cell microchamber that includes a Caco-2 monolayer, which are separated from each other by a nanoporous membrane (pore diameter of 50 nm). They further demonstrated that the individual transcriptional, metabolic and immunological profile of human epithelial cells cocultured with commensal *Lactobacillus rhamnosus*, are in line with in vivo data. The model is thus able to recreate a representative GI human–microbe interface that provides insight into linking the gut microbiome with human health or disease.

HuMix can also be useful for studying intestinal drug behavior. Specifically, this model might be suitable for investigating drug permeation and stability in one single setup. Noteworthy, however, is that the well differentiated Caco-2 layer showed significantly higher TEER values in the HuMix than in a transwell-device, possibly influencing drug permeation. For a more in depth description of the system, we refer to [6].

##### 6.2.4.4. Intestine-Chip with Microbiome

Besides small and large intestinal chips, several MPS have been established that enable simultaneous aerobic and anaerobic culturing conditions. These chips can incorporate a microbiome component with either Caco-2 cells or small intestinal organoids. However, they are currently not characterized with respect to drug disposition [202,231,232].

**Figure 14:**
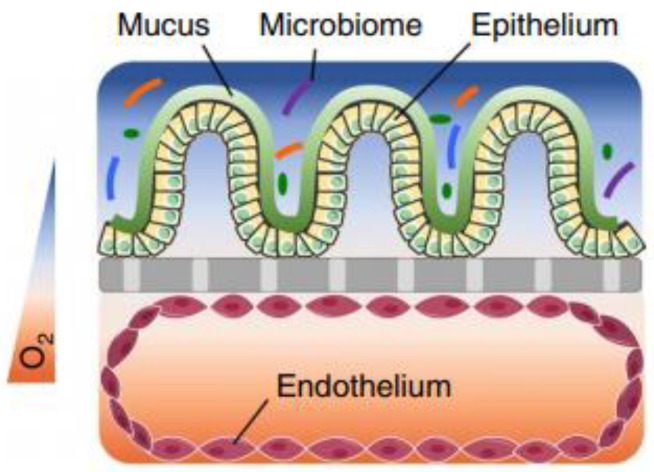
Representation of the microfluidic intestine-chip with microbiome, under the presence of an oxygen gradient (color scale). The human intestinal epithelium is overlaid with its own mucus layer and complex gut biota and positioned over an extracellular matrix-coated porous membrane (grey). The vascular endothelium is situated below the porous membrane. Adopted with permission from [231], Springer Nature, 2019.

Extended coculturing of aerobic and anaerobic commensal bacteria with intestinal epithelial cells using chip technology is described by Jalili-Firoozinezhad and coworkers [231]. A transluminal hypoxia gradient is set in place in a microfluidic intestine-on-a-chip that achieves low oxygen-concentration (<0.5%) by using an oxygen-sensing, dual channel chip. The chip comprises epithelial cells that are grown on a PDMS polymer that face an anaerobic chamber on the top and are supported by an oxygen and medium containing flow-channel on the bottom, which is lined by endothelial cells (Figure 14). The intestinal chip has been developed with increments in physiological complexity. Initially Caco-2 cells were used as an epithelial source, and cocultured with one anaerobic strain (*Bacteroides fragilis*). Interestingly, the P*_app_* of Cascade blue was reduced by approximately 2-fold in the presence of the anaerobic bacteria compared with the aerobic conditions, indicating an increased barrier integrity, which is in line with previous data in a Caco-2 coculture chip [202]. Moreover, *Bacteroides fragilis* could be detected on top of an approximately 10 µm thick mucus layer excreted by the Caco-2 cells. Next, the authors used complex gut microbiome that was isolated from human feces and then maintained in gnotobiotic mice for over 30 generations. The number of operational taxonomic units after 3 days of coculturing was at a similar scale as in human intestinal aspirates, indicating that an intact, commensal microbiome with aerobic and anaerobic strains could be maintained. Finally, intestinal organoids isolated from the nondiseased regions of surgical ileal biopsies from a 15-year-old UC patient were cocultured with commensal bacterial from fecal samples from preterm infants (born before 32 weeks of gestation). The coculture could be sustained for at least 5 days. The primary epithelial cells formed a polarized epithelium with villin-containing brush boarders and MUC2 producing cells. The presence of secreted mucus was confirmed by Alcain-blue staining, and similarly to the Caco-2 conditions, the presence of the microbiome did not negatively impact the viability of the epithelial cells in this in vitro setting, with no toxic effect or invasion of certain strains into the epithelium reported. In fact, it is striking that the presence of commensal bacteria consistently leads to increased barrier integrity, as demonstrated by either an increase in TEER and/or a decrease in the P*_app_* value of high molecular integrity markers [202,231,232].

The physiological chip described by Jalili-Firoozinezhad and coworkers with built-in oxygen gradient holds great potential to assess drug disposition under colon-emulating (currently the chip is derived from the ileum) conditions but seems to require a complex experimental setup (controlling different chip conditions) and can only be used at low throughput rate.

## 7. Concluding Remarks and Future Perspectives

Over the past 20–30 years, IBD cases have increased substantially [233]. Similarly, CRC incidence is continuously rising, especially in developing countries, with CRC being the third leading cause of cancer death and the fourth most commonly diagnosed cancer in the world [234]. Considering the need for long-term therapies, drugs with a high colonic exposure but low systemic exposure thereby minimising adverse effects, are most desired for treatment of IBD and CRC. Therefore, different colon-targeted drug delivery systems have been developed and various strategies are currently being used in formulations. To achieve successful colon targeting, several colon-specific hurdles like the luminal contents (low water volume, high viscosity, dynamic stress, variable composition etc.), the presence of a thick mucus lining, an increased barrier tightness compared to the small intestine, and the presence of a metabolically active and patient-specific microbiome need to be addressed and overcome. Currently, in vitro tools fully representing these colon-specific features that can aid in the development of drugs with a favorable profile are limited and, therefore, colon-targeting formulations suffer from inconsistent performance. The successful development of colon-targeting formulations for which efficacy and safety can be guaranteed (despite substantial intra- and interindividual patient differences), requires a thorough understanding of the colon-specific physiology and a tool set that can emulate the colonic environment. However, there has been promising progress in the field. Different models and setups have been developed aiming to mimic GI and colonic physiology and environment, as it is challenging to develop one model representing all in vivo parameters. Media have undergone increments in complexity in order to adequately assess colonic drug behavior in vitro, ranging from simple physiological buffers and more complex biorelevant media to rat cecal contents and human fecal contents that resemble the in vivo situation more closely. With the recent advances made over the past 5–10 years in culturing technologies and in the bioengineering of stem cell-derived systems, the availability of innovative intestinal systems more closely resembling the in vivo environment than traditionally used models like Caco-2 has also increased substantially. Although the characterization with respect to the absorption and metabolism properties of those stem cell-derived, bioengineered platforms and organ chips has been so far mainly focused on the small intestinal regions, it is obvious that the same technologies can be applied for the colon. Despite differences in cell type ratios, tight junctions, water volume, and pH, the most crucial differences between the small and large intestine to be captured with an in vitro tool are the presence of a thick bilayered mucus and commensal bacteria in the colon. Efforts towards a colonic system enabling simultaneous permeability and stability assessments of drugs seem to be on the way or already achieved, but still at low throughput capacity and not characterized for the use in permeability and metabolism studies (Intestine-Chip with microbiome, HuMix) [6,202,231].

Table 3 and Table 4 provide an overview of the characteristics of main intestinal in vitro tools discussed here, categorized into epithelial and microbiome platforms. Moreover, when further assessing those novel systems in their capability to accurately capture colonic drug disposition, one should consider the importance of accumulation studies in colonic systems. This is specifically crucial for colon-targeted drugs since they often aim to directly target the epithelium or immune cells of the lamina propria and are actually less desired to enter the blood stream.

In summary, over the past decade a large number of novel organotypic small intestinal and colonic models have been developed and used in molecular biology, regeneration studies, and disease modelling. Despite closer resembling the intestinal microphysiology, the flexibility which many models provide with regards to intestinal regions and donor populations (different age, gender, healthy, diseased, etc.) can be beneficial to assess the ADME properties of drugs in a patient-oriented manner. However, further characterization and direct comparison of small intestinal and colonic models with regards to drug permeation and metabolism is needed to understand the added value of their increased complexity in drug disposition.

## Figures and Tables

**Table 1:** Schematic overview of oral colon-targeted drug delivery systems.

Oral Colon-targeted Drug Delivery Systems	Mechanisms
Currently available systems	- pH-dependent release - Time-dependent release - Multimatrix system - Bacterial degradation (enzymatic)
Investigational systems relying on (patho)physiology	- Size-dependent nanodelivery systems - Mucoadhesive system
	- Pressure-controlled release - Osmotic-controlled release

**Table 2:** Composition of simulating the distal ileum (SIF_ileum_/SIF_ileum_-V2), ascending colon in the fasted state (FaSSCoF) and in the fed state (FeSSCoF/FeSSCoF-V2).

	SIF_ileum_ [132]	SIF_ileum_-V2 [131]	FaSSCoF [131,132]	FeSSCoF [132]	FeSSCoF-V2 [131]
Sodium cholate (mM)	0.8	-	0.15	0.6	0.6
Lecithin (mM)	0.2	-	0.3	0.5	0.5
Sodium oleate (mM)	-	-	0.1	0.2	0.2
Glucose (mg/mL)	-	-	-	14	4.8
Maleic acid (mM)	52.8	120	75.8	30.15	65
Tris (mM)	-	-	45.4	30.5	65
NaOH (mM)	105	240.6	120	16.5	16.5
Sodium chloride (mM)	30.1	-	-	34	-
pH	7.5	8	7.8	6.0	6.0

**Table 3:** Overview of the characteristics of intestinal in vitro tools (epithelial models) discussed in this article.

In Vitro System.	Source	Regions	Apical & Basal Side	Preclinical Species	Drug Permeability Data (Human)	Drug Transporter (Human)	Drug Metabolising Enzymes (Human)	Through-Put	Long-Term Viability
Caco-2 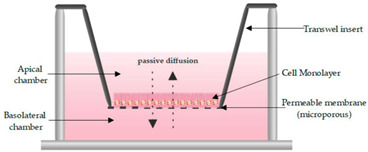 Figure 2	Colorectal adeno-carcinoma [1]	Colon-derived but small intestinal-like [1]	Yes [1]	No	Large data set [162,235]	mRNA [235], and protein [155,236] data; functionally characterized [1,237,238]	mRNA [239,240], and protein data [236]; less functionally characterized due to lack of CYP3A4 [157,241]	Medium–high	Standard 21 days for differentiation, up to 7 days afterwards [1]
Ussing Chamber 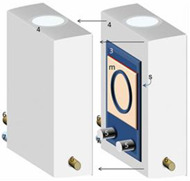 Figure 3	Primary intestinal tissue [185]	All regions possible [185]	Yes [185]	Possible	Large data set [175,180]	mRNA, and protein data (tissue) [27,28]; functionally characterized [242]	mRNA, and protein data (tissue); limited functional characterization [241,243,244]	Low	No (several hours) [185]
Organoids 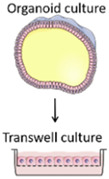 Figure 4	Primary intestinal tissue [49,187,188]	All regions possible [49,187,188]	Possible [49,197]	Yes [187,245,246]	Not characterized	Very limited mRNA, and protein data; very limited functional characterization [195]	Very limited mRNA data; very limited functional characterization [195]	Medium–high	In differentiated conditions 1 week [185]
EpiIntestinal^TM^ 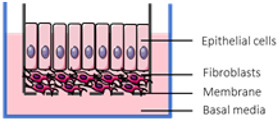 Figure 7	Primary intestinal tissue and fibroblasts [2]	Currently ileum [2]	Yes [2]	Currently Not	Basic characterization with different BCS class drugs [2,157]	mRNA [2], and limited protein [2] data; limited functional characterization [2,157]	mRNA [2]; functional characterization [2,157]	Medium–high	Up to 6 weeks [2]
Repligut^®^ 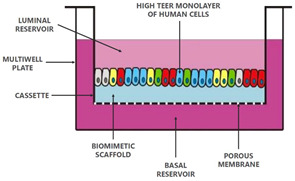 Figure 6	Primary intestinal tissue [208,247]	All regions possible, jejunum [247] and rectum [208] described	Yes [208,247]	Currently Not	Not characterized	No mRNA, and protein data; no functional characterization	No mRNA, and protein data; no functional characterization	Medium–high possible	In differentiated conditions 1 week (company information)
3D-bioprinted small intestinal tissue 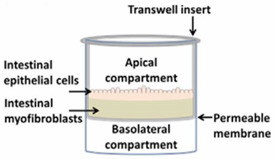 Figure 8	Primary intestinal tissue and fibroblasts [3]	Currently ileum [3]	Yes [3]	Currently Not	Very basic characterization with limited number of high and low permeable drugs [3]	mRNA [3] data; limited functional characterization [3]	mRNA data [3]; limited functional characterization [3]	Medium–high possible?	Up to 3 weeks [3]
Organ Chip (Emulate) 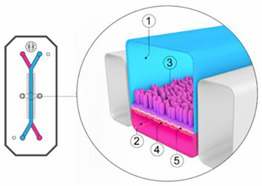 Figure 9	Primary intestinal and endothelial tissue [50,158]	Currently duodenum [158] and colon [50]	Yes [50,158]	Currently Not	Not characterized	mRNA [158] data; limited functional characterization [50]	mRNA [158] data; limited functional characterization [158]	Low–medium	2–3 weeks [158]
Primary enterocytes	Primary intestinal tissue [213,214,215]	All regions possible, all small intestinal regions described [215]	No [213,214,215]	Yes	Not characterized	No mRNA, and protein data; no functional characterization	mRNA [215]; functionally characterized [213,214,215,216]	High	Maximum 24 h (CHIM) [215]

**Table 4:** Overview of the characteristics of intestinal in vitro tools (microbiome models) discussed in this article.

Microbiome System	Bacterial Source	Coculture with Epithelial Cells	Apical & Basal Side	Preclinical Species	Drug Disposition Data (Human)	Through-Put	Coculture Viability
TIM-2 [218,219,220] 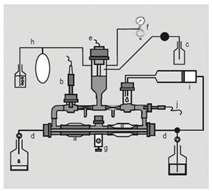 Figure 10	Feces from variable donors	No	No	Possible	Not characterized	Low	NA
SHIME^®^ [4] 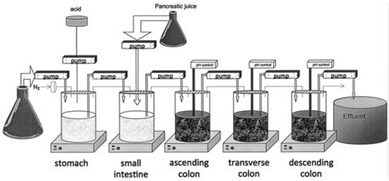 Figure 11	Feces from variable donors	No	No	Possible	Not characterized	Low	NA
HuMix [6] 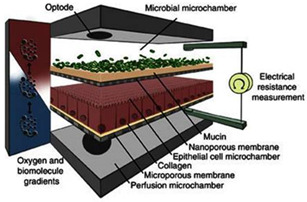 Figure 13	*Lactobacillus rhamnosus*	Caco-2 and primary epithelial cells	Yes	No	Not characterized	Low	
Intestine-Chip (Emulate) 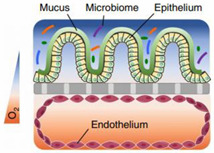 Figure 14	*Lactobacillus rhamnosus GG* [202]	Caco-2 [202]	Yes	No	Not characterized	Low	>than 1 week
	*Lactobacillus acidophilus, Lactobacillus plantarum, Lactobacillus paracasei, Bifidobacterium breve, Bifidobacterium longum*, and *Bifidobacterium infantis* [232]	Caco-2 [232]	Yes	No	Not characterized	Low	>than 1 week
	*Bacteroides fragilis* [232] and inoculation with feces from 4 different donors (infants) [232]	human intestinal organoids from 15-year old UC patient, noninflamed area, ileum [232]	Yes	No	Not characterized	Low	at least 5 days

## Abbreviations

ABC	ATP binding cassette
ADME(T)	absorption, distribution, metabolism, excretion (and toxicity)
ALI	air-liquid interface
API	active pharmaceutical ingredient
ASBT	apical sodium-dependent bile acid transporter
AUC	area under the curve
BCRP	breast cancer resistance protein
BCS	biopharmaceutical classification system
BSEP	bile salt export pump
CD	Crohn’s disease
CES	Carboxylesterase
CHIM^TM^	cryopreserved human intestinal mucosa
CRC	colorectal cancer
CYP	Cytochrome P450
DDI	drug–drug interaction
DME	drug metabolising enzyme
ECM	extra cellular matrix
F_a_	human fraction absorbed
FaSSCoF	fasted state simulated colonic fluid
FaSSIF	fasted state simulated intestinal fluid
FeSSCoF	fed state simulated colonic fluid
FeSSCoF-V2	fed state simulated colonic fluid version 2
FeSSIF	fed state simulated intestinal fluid
F_g_	human fraction escaping enterocyte metabolism
GI	Gastrointestinal
GLP	glucagonlike peptide
FITC dextran 4	fluorescein isothiocyanate dextran average molecular weight 4000
HIMEC	primary intestinal microvasculature endothelial cells
HT29-MTX	human colorectal adenocarcinoma cell line differentiated into mature goblet cells using methotrexate
HuMix	Human–Microbial crosstalk
IBD	inflammatory bowel disease
IC_50_	the half maximal inhibitory concentration
iPS	induced pluripotent stem cells
LGFR5	leucine-rich repeat-containing G-protein-coupled receptor 5
L-SHIME	Luminal Simulator of the Human Intestinal Microbial Ecosystem
MATE1	multidrug and toxin extrusion 1
MCT	monocarboxylate transporter protein
MMC	migrating motor complex
MMX	multimatrix
MRI	magnetic resonance imaging
MRP	multidrug-resistance-associated protein
M-SHIME	Mucosal Simulator of the Human Intestinal Microbial Ecosystem
MUC	Mucin
NAD	5-hydroxy-L-tryptophan and 5-hydroxytryptamin
NSAID	nonsteroidal anti-inflammatory drug
NTCP	sodium/taurocholate co transporting polypeptide
OAT2	organic anion transporting polypeptide 2
OATP	organic anion transporting peptide
OCT	organic cation transporter
OROS	osmotic controlled release system
P_*app*_	apparent permeability coefficient
PDMS	permeable polydimethyl siloxane
PEPT1	peptide transporter protein 1
P-gp	P-glycoprotein
qPCR	quantitative Polymerase Chain Reaction
SCFA	short chain fatty acid
SHIME	Simulator of the Human Intestinal Microbial Ecosystem
SIF_ileum_	simulating intestinal fluid
SIF_ileum_-V2	simulating intestinal fluid version 2
SLC	solute carrier
SLCO	Solute carrier organic anion transporter
SULT	Sulfotransferase
TEER	transepithelial electrical resistance
TIM	TNO Gastro-Intestinal Model
Tim-agc	TNO Gastro-Intestinal Model Advanced Gastric Compartment
T_max_	maximal plasma concentration
UC	ulcerative colitis
UGT	uridine 5′-diphospho-glucuronosyltransferases
ZO-1	zonula occludens 1
